# A Dehydration-Induced Eukaryotic Translation Initiation Factor iso4G Identified in a Slow Wilting Soybean Cultivar Enhances Abiotic Stress Tolerance in *Arabidopsis*

**DOI:** 10.3389/fpls.2018.00262

**Published:** 2018-03-02

**Authors:** Juan P. Gallino, Cecilia Ruibal, Esteban Casaretto, Andrea L. Fleitas, Victoria Bonnecarrère, Omar Borsani, Sabina Vidal

**Affiliations:** ^1^Laboratorio de Biología Molecular Vegetal, Instituto de Química Biológica, Facultad de Ciencias, Universidad de la República, Montevideo, Uruguay; ^2^Laboratorio de Bioquímica, Departamento de Biología Vegetal, Facultad de Agronomía, Universidad de la República, Montevideo, Uruguay; ^3^Unidad de Biotecnología, Instituto Nacional de Investigación Agropecuaria, Montevideo, Uruguay

**Keywords:** soybean, drought, translation initiation, abiotic stress, eIFiso4G, *Arabidopsis*

## Abstract

Water is usually the main limiting factor for soybean productivity worldwide and yet advances in genetic improvement for drought resistance in this crop are still limited. In the present study, we investigated the physiological and molecular responses to drought in two soybean contrasting genotypes, a slow wilting N7001 and a drought sensitive TJS2049 cultivars. Measurements of stomatal conductance, carbon isotope ratios and accumulated dry matter showed that N7001 responds to drought by employing mechanisms resulting in a more efficient water use than TJS2049. To provide an insight into the molecular mechanisms that these cultivars employ to deal with water stress, their early and late transcriptional responses to drought were analyzed by suppression subtractive hybridization. A number of differentially regulated genes from N7001 were identified and their expression pattern was compared between in this genotype and TJS2049. Overall, the data set indicated that N7001 responds to drought earlier than TJ2049 by up-regulating a larger number of genes, most of them encoding proteins with regulatory and signaling functions. The data supports the idea that at least some of the phenotypic differences between slow wilting and drought sensitive plants may rely on the regulation of the level and timing of expression of specific genes. One of the genes that exhibited a marked N7001-specific drought induction profile encoded a eukaryotic translation initiation factor iso4G (*GmeIFiso4G-1a*). GmeIFiso4G-1a is one of four members of this protein family in soybean, all of them sharing high sequence identity with each other. *In silico* analysis of *GmeIFiso4G-1* promoter sequences suggested a possible functional specialization between distinct family members, which can attain differences at the transcriptional level. Conditional overexpression of *GmeIFiso4G-1a* in *Arabidopsis* conferred the transgenic plants increased tolerance to osmotic, salt, drought and low temperature stress, providing a strong experimental evidence for a direct association between a protein of this class and general abiotic stress tolerance mechanisms. Moreover, the results of this work reinforce the importance of the control of protein synthesis as a central mechanism of stress adaptation and opens up for new strategies for improving crop performance under stress.

## Introduction

Cultivated soybean (*Glycine max* L.) is one of the top four global crops that, together with maize, rice, and wheat, produce nearly two-thirds of global agricultural calories (Tilman et al., 2011; Ray et al., 2013). Soybean worldwide is one of the major legume crops that provides oil and protein-rich food for both animal and human consumption. Moreover, cultivation of soybean is also important in agricultural systems due to its capability for fixing atmospheric nitrogen via symbiosis with rhizobia (Burris and Roberts, 1993) and therefore requires minimal input of nitrogen fertilizer. This species is commonly used in crop rotations to provide for residual fixed nitrogen to subsequent non-legume crops.

Water is usually the main limiting factor for soybean growth and productivity (Hufstetler et al., 2007; Fuganti-Pagliarini et al., 2017). In most soybean producing areas, intermittent drought is almost certain to occur during one or more stages of the plant’s growth cycle (Desclaux and Roumet, 1996). In temperate climates, it is frequent to encounter seasonal water constraints, particularly during summer, which may result in significant yield losses and reduction of seed quality (Meckel et al., 1984; Manavalan et al., 2009).

Genetic improvement for drought resistance has become an attractive but rather a difficult goal in soybean breeding programs. This is because drought resistance is a complex trait, which involves mechanisms that operate at different levels, for which there are limited genetic sources in the adapted breeding pool (Hufstetler et al., 2007).

Plants employ various mechanisms to cope with water deficit that can be classified into four groups: drought escape, drought avoidance, drought tolerance, and drought recovery (Carrow, 1996; Turner et al., 2001; Manavalan et al., 2009; Fang and Xiong, 2015). Drought escape allows plants to complete their life cycle before the occurrence of a serious water deficit period. The mechanism involves physiological and developmental characters that usually result in anticipated seed production through shortening of the life cycle. Drought avoidance is characterized by mechanisms that maintain high water potentials in plant tissues under mild or moderate water deficit conditions. Drought avoidance can be accomplished by adopting different strategies, such as increasing rooting depth, promoting an efficient root system, reducing stomatal conductance, leaf rolling or folding, reducing the evaporation surface, increasing wax accumulation on the leaf surface, enhancing water storage abilities in specific organs, etc. (Carrow, 1996; Manavalan et al., 2009; Fang and Xiong, 2015). All these strategies aim to increase the absorption efficiency by the roots, and/or to reduce the water loss by evapotranspiration from the aerial parts (APs) of the plants. Drought tolerance allows plants to continue metabolism even at low water potential. This mechanism involves a number of strategies, such as osmotic adjustment -in order to maintain cell turgor-, reactive oxygen scavenging and detoxification, adjustments to metabolism, regulated changes in plant growth, and other mechanisms to reduce or repair the damage resulting from the stress (Verslues et al., 2014; Fang and Xiong, 2015). Finally, drought recovery refers to ability of the plants to resume growth after exposure to a severe stress caused by water deficit (Luo, 2010; Fang and Xiong, 2015).

From all the mechanisms mentioned above, those related to drought escape may be useful for survival of a species in nature, but are generally not desirable in agricultural systems. However, sometimes drought escape can be achieved artificially without yield penalty, by adjusting the growth period or by choosing crop varieties with short life cycles, to prevent local seasonal or climatic drought (Manavalan et al., 2009; Fang and Xiong, 2015). On the other hand, traits associated with drought avoidance and tolerance are generally interesting for breeding for drought resistance. Some of these traits, particularly those related with drought avoidance mechanisms, can be associated with the modulation of the root system architecture. However, several other traits have to do with the AP of the plant and they usually involve mechanisms of both drought avoidance and tolerance (Fang and Xiong, 2015). Some of the leaf traits associated with drought avoidance are related with the phototropic movement of leaves in response to water deficit, which regulate the interception of solar radiation and thus water loss through the leaves (Meyer and Walker, 1981; Ludlow and Bjorkman, 1984). Thus, plants having erect leaves under water deficit conditions will receive less radiation and control better their water content. Other drought resistant traits that are associated with the AP of the plant have to do with morphological features of the leaves that tend to reduce water loss and to enhance the water holding ability of the plant under stress (Fang and Xiong, 2015). Examples of these are the presence of smaller and thicker leaves, thicker cuticle, smaller and more abundant stomata, more trichomes, among others.

When plants sense severe water deficit, their leaves wilt due to the loss of cell turgor pressure (Poorter and Markesteijn, 2008). Canopy wilting is the first visible symptom of stress caused by water deficit in soybean (King et al., 2009). During the last two decades of the late 20th century, several plant introductions (PIs) exhibiting slow wilting trait under water deficit conditions, were discovered and used in soybean breeding programs (Carter et al., 1999). Soybean plants showing slow wilting phenotype have been extensively studied by different groups (Charlson et al., 2009; King et al., 2009; Sadok and Sinclair, 2009; Abdel-Haleem et al., 2011; Ries et al., 2012; Sadok et al., 2012; Devi and Sinclair, 2013; Pathan et al., 2014). One of these genotypes, the PI 416937 was shown to have several root related traits associated with drought avoidance, such as a highly prolific root system (Pantalone et al., 1996), and an increased lateral root growth (Hudak and Patterson, 1996). However, PI 416937 stands out over other genotypes also due to leaf related traits, which are associated to its capacity to limit transpiration in response to vapor pressure deficit (Sloane et al., 1990; Fletcher et al., 2007). It is unknown whether slow wilting involves a single mechanism of drought resistance or may be the result of the integration of several different mechanisms (Hufstetler et al., 2007). Therefore, the molecular and physiological bases for this trait and other drought resistant traits should be better understood in order to be useful in breeding programs.

In this study, we analyzed the molecular and physiological responses of two soybean genotypes showing contrasting phenotypes under water deficit conditions. One of these genotypes, the cultivar N7001, is a stabilized offspring of a cross between a cultivar and the slow wilting PI 416937 (Carter et al., 2003). The other genotype used in this study is the cultivar TJS2049, which is considered to be highly sensitive to water deficit (Pardo et al., 2014). The results provide an insight into the drought resistance mechanisms of two contrasting cultivars, one of them exhibiting slow wilting phenotype.

Using suppression subtractive hybridization (SSH), a cDNA library enriched in drought-induced genes from N7001 was constructed and used to identify genes that were differentially regulated upon drought stress between N7001 and TJS2049. This approach was chosen over others due to its high reproducibility, low false positive rate, relatively low cost, and most importantly, because it is a powerful method to identify low abundant transcripts (Diatchenko et al., 1996; Huang et al., 2007; Zhang et al., 2012).

We identified stress-related genes that were differentially expressed between the two genotypes in response to water deficit. One of the genes that was found to be upregulated in N7001 but not in TJS2049, coded for a eukaryotic translation initiation factor eIFiso4G (designated here GmeIFiso4G-1a). In this work, we assessed the function of *GmeIFiso4G-1a* in plant stress tolerance by the ectopic conditional overexpression of the gene in *Arabidopsis*. Transgenic plants exhibited increased osmotic, salt, drought and low temperature stress tolerance, providing an experimental evidence for a direct involvement of a translation initiation factor from this class in abiotic stress tolerance.

## Materials and Methods

### Plant Material and Growth Conditions

The soybean genotypes used were N7001 (Carter et al., 2003) and TJS2049. Soybean plants were grown in a growth chamber with a 16/8 h (light/darkness) photoperiod, temperatures of 30 and 20°C for day and night, respectively, and an irradiancy regime of 800 μmol m^-2^ s^-1^ using metallic halogen lamps (400 W) and sodium incandescent lamps (75 W). The light from the growth chamber was supplemented with an input of natural sunlight.

Plant pots consisted of PVC cylinders of 11 cm diameter and 30 cm high, having the bottom covered with a metal mesh. Tubes were filled with a mix of sand/vermiculite (ratio 1/1) as substrate. Initially, three soybean plants were grown per pot, but once plantlets were established, only one plant was chosen and left in each pot to continue growing. Plants were watered daily to field capacity, with Rigaud and Puppo (1975) medium supplemented with 10 mM of KNO_3_ through a holed tube. In order to attain maximum water retention capacity of the substrate, pots were watered up until excess of water drained from the bottom of the pots through the metal mesh. The pots were kept in this condition for 24 h, until no water excess drainage was observed. In this way, the maximum volume of water held by the substrate was quantified and the resulting value was used as reference to express the percentage of water retained by the soil substrate during drought stress.

*Arabidopsis thaliana* accession Columbia glabrous, alias Col-5 (*gl1*) was used in this study. Plants were grown *in vitro* by surface sterilizing their seeds with 7% of bleach with 0.05% Tween-20 and exposing them for 3 days to 4°C for stratification before placing them in Petri dishes. Plants were grown in half strength MS medium [2.4 g/L Murashige and Skoog, 0.5 g/L 2-(N-morpholino) ethanesulfonic acid hydrate and 0.5% phyto agar, pH 5.7], at 22°C with a 16/8 h day/night cycle and a photon flux of 120 μmol photons m^-2^ s^-1^.

*Arabidopsis* plants were also grown in pots or perforated 50 ml falcon tubes, filled with a mixture of moss peat and vermiculite (1:1). Seeds were germinated in sterile conditions on MS plates, transferred to pots or tubes (covered with a plastic dome during 3 days), and allowed to grow at 22 °C, under a 16/8 h (light/darkness) regime, using MS for irrigation.

### Soybean Drought Stress Conditions

Soybean plants were grown for 35 days, until they reached the V5 developmental stage, unless otherwise stated. During this period, plants were grown in substrate irrigated at maximum substrate water retention capacity. Experimental replicas of three pots per genotype and per treatment were used, and the replicas were randomly distributed in the growth chamber to rule out possible variations due to small differences in environmental conditions that may have occurred in different locations within the growth chamber.

Plants were subjected to drought stress after 35 days of growth when they reached the V5 stage. At that moment dehydration stress was conducted by terminating irrigation, and left until 25% of the maximum substrate water retention capacity (substrate water potential ψ = ∼2.2 MPa). Non-stressed control plants at the V5 stage continued to be daily irrigated at maximum substrate water retention capacity (100%). Samples were taken when substrate reached a 50% water retention capacity (ψ = ∼0.7 MPa) to evaluate the early response at moderate stress conditions, or when substrate reached 25% of the water retention capacity, to monitor the late plant response, for severe stress conditions.

### Physiological Parameters Used for Monitoring Drought Stress Status of Soybean

#### Water Content of Substrate

Substrate water content was measured by determining the field capacity (FC), which represents the water holding capacity of the substrate. We considered a maximum FC of 100% as the situation when the substrate was unable to retain additional water and therefore water ran off the substrate. Water substrate content was determined by measuring the substrate weight under drought stress and under irrigation conditions.

#### Stomatal Conductance

Stomatal conductance was measured with a Porometer Model SC-1 (Decagon Device), on the abaxial surface of leaves, as instructed by the manufacturer. Three foliates per plants and three measures per leaf were analyzed. The leaves analyzed were at the same phenological stage.

#### Leaf Water Potential

Leaf water potential of the third trifoliate was determined with a Scholander type pressure pump, Model 600 (PMS Instrument Company) according to manufacturer’s instructions.

#### Water Use Efficiency (WUE)

Plants in V4 developmental stage were used for determinations of WUE by the gravimetric method. Plants were grown on substrate at 100% of their maximum capacity water retention (Control) and at 35% of its maximum water retention capacity (Stress), with four repetitions for each treatment. The 1st day of stress the accumulated dry matter (DM) of the AP was determined in four repetitions of each genotype. For 18 days after the imposition of stress, water consumption was daily recorded and the water consumed was recovered. The accumulated DM of AP was then evaluated in both genotypes in the different treatments. The DM was determined by drying the samples at 105°C until constant weight.

The WUE was calculated as:

$$WUE(g/kg)=\frac{\text{Final DM of AP }\left(\text{gr/pot})-\text{Initial DM of AP }(\text{gr/pot}\right)}{\text{Water consumed}}$$

#### Carbon Isotope Ratio (∂^13^C‰)

One and a half mg of the dried tissue samples used for DM determinations, were placed in tin capsules to determine the amount of ^13^C and ^12^C in an elemental analyzer (Flash^®^ EA, 1112 Series) coupled to an isotope ratio mass spectrometer (Thermo Finnigan Delta Plus^®^). The *R*-value (^13^C/^12^C molar abundance ratio of the sample) is expressed in relation to the Pee Dee Belemnite standard (PDB). The values of ∂^13^C ‰ were obtained through comparison of ^13^C/^12^C molar abundances of leaf samples from the different treatments with respect to the PDB standard according to O’Leary (1993):

$$\partial^{13}C(‰)=[\left(R_{sample}/RPDB\right)-1]\times 1000‰,$$

where ∂^13^C is the ratio of carbon isotopes, *R*_sample_ is the ratio of the ^13^C/^12^C molar abundance of the plant sample and RPDB is the ratio of the Standard ^13^C/^12^C molar abundance (PDB), which is equal to 0.0112372 (Squeo and Ehleringer, 2004).

#### Relative Water Content (RWC)

The RWC was determined according to Antolín et al. (1995). Fresh leaves were weighed (FW) thereafter placed in 20 cm Petri dishes containing distilled water, and incubated at room temperature for 12 h after what leaves were reweighed to obtain their weight saturated (*W*_sat_) value. For dry weight (DW) determination each sample was dried in an oven at 80°C until constant weight was attained. The RWC was calculated using the formula: RWC: [(FW - DW)/(*W*_sat_ - DW)] × 100.

### Antioxidant Enzyme Determination, Proline Content and *in Situ* Detection of ROS in Soybean

Soybean samples were taken at V3 stage, from well-watered plant controls (100% FC), and at 50% or 25% FC. All biochemical parameters were measured using leaf material from the second trifoliate. Four plants at the same phenological stage were analyzed for proline content and antioxidant enzyme activities.

Proline was extracted from 100 mg of plant tissue with a mixture of methanol-chloroform-water (12:5:1) as described by Charest and Phan (1990) and quantified according to Borsani et al. (1999).

Catalase (CAT), Ascorbic peroxidase (APX) and superoxide dismutase (SOD) activity determinations were carried out using 200 mg of fresh material, homogenized with 2 mL of extraction buffer (0.1 M potasium phosphate buffer pH 7.0, 1 mM EDTA, 0.2% ascorbic acid, 0.1% triton 100X, 15% glycerol, 1% PVP, 1 mM PMSF; 0.36 μM β-mercaptoethanol) and centrifuged 15000 *g* at 4°C for 10 min. Supernatants were used for all enzymatic determinations. Total proteins were quantified by Bradford.

Catalase and APX kinetic reactions were performed in 1 mL quartz cuvettes containing 990 mL of 50 mM potassium phosphate buffer pH 7.0, 10 mM H_2_O_2_ and 10 μL of extract for CAT, or 950 mL of 50 mM potassium phosphate buffer pH 7.4, 10 mM H_2_O_2_, 0.5 mM ascorbic acid and 20 μL of extract for APX, as described by Signorelli et al. (2013). The kinetics were done by measurement of H_2_O_2_ (Aebi, 1984) or ascorbic acid decay (Hossain and Asada, 1987) in 90 s at 240 nm or 290 nm, respectively. All measurements were performed at least three times per sample. Enzyme activities were expressed in units per mg of protein (U/mg).

Cytosolic and chloroplastic SOD isozymes were analyzed in non-denaturing polyacrylamide gels using in-gel activity assays with isozyme-specific inhibitors previously described by Donahue et al. (1997). 20 μg of proteins were loaded in non-denaturing gels (12% acrylamide) and run for 2 h at 30 mA at 4°C. One gel was incubated during 30 min in dark using the reaction buffer without inhibitors (50 mM potasium phosphate buffer pH 7.4, 1 mM EDTA, 0.24 mM nitro blue tetrazolium (NBT), 33.2 μM riboflavin, 0.2% TEMED). A second gel was incubated in reaction buffer containing 2 mM KCN for the inhibition of Cu-Zn-SOD, and the third gel was incubated in reaction buffer containing 5 mM H_2_O_2_ for inhibition of Cu-Zn-SOD and Fe-SOD. After dark incubation, the gels were exposed to light and photographed.

*In situ* detection of superoxide or H_2_O_2_ was performed according to Jabs et al. (1996) or to Thordal-Christensen et al. (1997), respectively. Soybean leaf disks were vacuum infiltrated with 10 mM potassium phosphate buffer pH 7.8, 10 nM NaN3, 0.05% (v/v) Tween 20, containing 1 mg/ml NBT for superoxide detection, or containing 1 mg/ml of 3.3-diaminobenzidine (DAB) in water adjusted to pH 3.8 with HCl for H_2_O_2_ detection. Negative control for DAB staining was performed using water, instead of DAB solution, adjusted to pH 3.8 with HCl. The infiltrated leaves were kept for 30 min under daylight conditions prior to ethanol bleaching.

### Construction of Subtractive Libraries

A suppression subtractive hybridization library, enriched in drought-induced genes from the soybean genotype N7001, was generated using PCR Select-cDNA Subtraction Kit (BD Biosciences Clontech), and cloned into Invitrogen pCR-II TA cloning vector. Total RNA was extracted from leaf samples from N7001 genotype at V5–V6 stage, of drought stressed plants and of non-stressed control plants, at time points corresponding to 3 days (early response) and 7 days (late response), after the onset of drought stress. Leaflets from each trifoliate leaf, from three replica plants were pooled to constitute one sample. RNA was extracted from each sample using standard procedures based on phenol/chloroform extraction followed by LiCl precipitation. Fifty μg of total RNA extracted from plants sampled at each time point were pooled together, thereby generating a single total RNA sample corresponding to drought-stressed N7001 plants, and a single total RNA sample corresponding to non-stressed N7001 control plants. mRNA was then purified from total RNA samples using the kit *MicroPoly (A) Purist* (Ambion, Inc., Austin, TX, United States), and 2 μg of mRNA was used to synthesize cDNA for the suppression subtractive hybridization procedure using the PCR Select-cDNA Subtraction Kit (BD Biosciences Clontech). The library was constructed using the samples derived from drought-stressed plants (DS) as tester and the samples derived from non-stressed control plants as driver. The resulting secondary PCR products from the forward subtracted library were directly cloned into the pCR-II plasmid vector, and transformed into *Escherichia coli* TOP 10 competent cells, using the TA cloning kit Dual Promoter from Invitrogen (Life Technologies). Approximately 1000 white putative recombinant colonies were selected, purified and analyzed for the presence of insert DNA. Plasmid DNA was isolated for 800 insert containing clones and sequenced using SP6 primer.

### Sequence Analysis of cDNA Clones

The gene ID of each clone was identified by BLASTn comparisons vs. the genome of *G. max*, cv William 82, available at Phytozome database https://phytozome.jgi.doe.gov/pz/portal.html#!info?alias=Org_Gmax (accessed November 2017). Functional annotation was determined by BLASTx comparisons (*E*-value < 10^-5^) vs. Phytozome and GenBank protein databases. Genomic sequence of *GmeIFiso4G-1a* from N7001 cultivar was deposited in the GenBank with the accession number of MG902957.

### Differential Screening of Macroarrays by Reverse Northern Blots

Reverse Northern blot analysis of differentially expressed sequences present in the subtracted cDNA library were carried out using the PCR-Select^TM^ Differential Screening Kit (BD Biosciences Clontech). Fifty ng of insert-derived PCR-amplified fragments of the library clones were dot-blotted onto Nylon membranes and probed with α ^32^P dCTP -labeled subtracted probes. DNA samples corresponding to individual bacterial clones were arrayed in groups of 96 and blotted 8 times for hybridization with eight different subtracted probes.

The probes derived from RNA samples obtained from the drought-tolerant N7001 soybean variety or from the drought-susceptible TJ2049 soybean variety. RNA samples were obtained at time points corresponding to approximately 3 days of stress (50% FC), for monitoring the early response), or 7 days after the onset of drought stress (25% substrate capacity), for monitoring the late response. Samples from non-stressed control plants were taken at the same time points. For each plant variety, the following four probes were generated: (i) Forward – early response subtracted probe: the samples derived from 3 days DS was used as tester and the sample derived from non-stressed control plants was used as driver; (ii) Forward – late response subtracted probe: the sample derived from 7 days DS was used as tester and the samples derived from non-stressed control plants was used as driver; (iii) Reverse early control subtracted probe: control sample was used as tester and 3 days stress sample was used as driver; (iv) Reverse late control subtracted probe: control sample was used as tester and 7 days stress sample was used as driver. The resulting secondary PCR products were labeled with [α-^32^P] dCTP according to kits instructions, and purified using CHROMA SPIN-100 (BD Biosciences Clontech). Hybridizations with subtracted probes were done according to PCR-Select^TM^ Differential Screening Kit’s protocol. Hybridizations were done using *Express Hyb blotting solution* (BD Biosciences Clontech), washed twice with 2X SSC, 0.05% SDS and twice with 0.1X SSC, 0.1% SDS. Membranes were analyzed in a Fuji Image Analyzer FLA-3000.

### Northern Blots

Soybean cDNA-inserts of the selected clones were used as probes to confirm the differential expression of the corresponding soybean genes by Northern blot. For this, total RNA was isolated from drought stressed and from non-stressed control plants (Ctrl) of soybean N7001 and TJ2049 cultivars, using standard procedures. Plants were exposed to water deficit and sampling was performed when FC reached 50%.Ten μg of total RNA were separated in denaturing formaldehyde agarose gels and transferred onto nylon membranes (Hybond XL, Amersham Pharmacia Biotech). Ethidium bromide staining of the gels was used to ensure that equal amounts of RNA were loaded in the gels. Membranes were prehybridized at 65°C in 5X SSPE, 5X Denhardt’s solution, 0.2% SDS and 0.5 mg mL^-1^ denatured salmon sperm DNA, and hybridized overnight at 65°C with [α-^32^P] dCTP labeled probes. Fifty ng of DNA from purified cDNA insert clones were labeled using Amersham Rediprime II DNA Labeling System (GE Healthcare Life Sciences) and used as hybridization probes. The sequences spanning the positions in the CDS of the genes used as probes were the following: nt. 2361 to nt. 2653 (Phototropin 2); nt. 159 to nt. 449 (Histone 2A); nt. 2341 to nt. 2857 (eIFiso4G); nt. 133 to nt. 429 (MBF1-like transcription factor); nt. 1759 to nt. 2004 (GT-2 transcription factor); nt. 1 to nt. 599 (AN1-like Zinc finger); nt. 683 to 1007 (Nucleoredoxin); nt. 71 to nt. 342 (Dehydrin-13 kDa); nt. 388 to nt. 762 (Dehydrin-27 kDa); and nt. 2 to nt. 269 (Glutathione peroxidase).

In the case of *GmeIFiso4G-1a*, the full-length cDNA sequence was also used as a probe for mRNA detection in transgenic *Arabidopsis*. After hybridization, filters were washed twice for 30 min at 65°C with 5X SSC-0.5% SDS and twice using the same conditions with 1X SSC-0.5% SDS. Membranes were exposed to autoradiography films at -86°C and developed.

### Phylogenetic Analysis

Translated protein sequences from soybean *eIFiso4G* and *eIF4G* and *Arabidopsis eIFiso4G* genes were retrieved from Phytozome database^1^. Sequences were aligned with ClustalW and phylogenetic analysis were done using MEGA 6 software (Thompson et al., 1997; Tamura et al., 2011). Phylogenetic tree was done using Neighbor joining and Maximum likelihood methods, both of them showing similar results.

### Constructs for Overexpression

The construct for conditional overexpression of *GmeIFiso4G-1a* was generated by cloning the cDNA coding region of the gene, into pENTR-2B vector (Invitrogen-Thermo Scientific) as an *Sal*I/*Eco*RI fragment. One μg of total RNA extracted from drought-exposed N7001 plants was reverse transcribed into cDNA using oligo(dT) primer and SuperScript II Reverse Transcriptase (Invitrogen, Thermo Fisher Scientific). The coding region of *GmeIFiso4G-1a* was PCR amplified using *Pfu* DNA polymerase (Thermo Scientific) and the primers, F: 5′-ACGTCGACGATTGCATCGCGAGGTATTA-3′ and R: 5′-CGGAATTCTCATGCAAACTCT CATCTGATTC-3′, containing *Sal*I and *Eco*RI restriction sites, respectively. The PCR fragment was cleaved with restriction enzymes and ligated into pENTR-2B. The resulting clones were fully sequenced and further recombined into *att*R sites of pMDC7 binary vector (Curtis and Grossniklaus, 2003) using Gateway LR Clonase II (Invitrogen-Thermo Scientific). The construct was introduced into *Agrobacterium tumefaciens*, strain C58C1 (Deblaere et al., 1985) by electroporation.

### *Arabidopsis* Transformation and Molecular Characterization of Transgenic Lines

Transgenic *Arabidopsis* plants were produced by *Agrobacterium*-mediated floral-dip method (Clough and Bent, 1998), and identified by PCR with specific primers for *GmeIFiso4G-1a*. Expression of the transgenes was tested in 12 independent T2 lines by northern hybridization after treatment of plants with 5 μM β-estradiol (Sigma-Aldrich, E2758). Homozygous transgenic T3 plants were produced from single T-DNA insertion lines, selected by 3:1 segregation of hygromycin resistance. Transcript levels of two selected independent T3 lines (OE-5 and OE-8) were confirmed by semi quantitative (RT)-PCR in the presence or absence of β-estradiol, using *GmeIFiso4G-1a* specific primers. (F: 5′-ACGTCGACGATTGCATCGCGAGGTATTA-′3 and R: 5′-CGGAATTCTCATGCAAACTCT CATCTGATTC-′3), giving rise to a 2370 bp amplicon that corresponded to the complete cDNA sequence of the gene. The PCR was performed using an annealing temperature of 56°C and 30 cycles, with an extensión time of 3 min. *Arabidopsis* ubiquitin gene (At4G05320) was employed as an internal control for constitutive expression, the primers F: 5′-ACCGGCAAGACCATCACTCT-′3, and R: 5′-AGGCCTCAACTGGTTGCTGT-′3, and PCR conditions of 55°C as annealing temperature, and 25 cycles.

### Stress Conditions and Phenotypic Evaluation of *Arabidopsis*

*Arabidopsis* wild type or transgenic lines were grown *in vitro* or in substrate and exposed to salinity, osmotic stress, low temperature (4°C) or dehydration. In all experiments, the phenotype of *Arabidopsis* plants was analyzed in the presence or absence of 5 μM β-estradiol. Treatment with β-estradiol to *in vitro* grown plants, was done by incorporating this compound onto the growth medium, and kept during the time that the stress was imposed. *In vitro* grown wild type or transgenic *Arabidopsis* were exposed to salt stress by transferring to 5-day-old seedlings to square Petri dishes containing MS supplemented with 150 mM NaCl. Osmotic stress was imposed by transferring 5-day-old *in vitro* grown seedlings to 300 mM mannitol or 40% polyethylene glycol (PEG) 8000-infused agar plates (square), made according to Verslues et al. (2006). For all treatments, plates were placed vertically and plant growth was monitored during 9 days of stress by measuring root weight and monitoring root elongation. Twenty plants were individually measured for each time point, and averages were calculated. Fresh weight (FW) and DW were also measured for each plant and averaged. Experiments were repeated three times.

Drought stress of *Arabidopsis* wild type or transgenic lines was performed in non-sterile conditions using plants germinated in pots and transferred to 50 ml falcon tubes with peat/vermiculite (1/1), when the cotyledons were fully expanded. Plants were watered for 10 days with half strength MS medium and subsequently, dehydration was performed by interrupting irrigation for 9 days. Treatment with β-estradiol was done by adding every 24 h, 200 μL of 5 μM β-estradiol on top of the center of the rosette for during the time that the stress was imposed. Controls were treated in the same way by adding identical volume of water with the addition of equal amount of DMSO instead of β-estradiol. Three biological replicates were done, each of them containing 10 plants per treatment. After harvesting, samples were immediately placed in liquid nitrogen and stored at -80°C until further analysis.

Cold treatment was performed to 10-day-old wild type and transgenic lines, grown together on pots with a diameter of 15 cm and then exposing the plants to 4°C. Plants were exposed to low temperature for 2 weeks, with a 16-h-light/8-h-dark light regime and 120 μmol photons m^-2^ s^-1^. Three biological replicates were done, each of them containing 10 plants per treatment.

For proline or anthocyanin determination, four plants per sample were powdered using liquid N_2_ and stored at -80°C until processing. Proline was extracted from 100 mg of plant tissue as described above and anthocyanin content was determined according to Neff and Chory (1998). Briefly, 100 mg of plant tissue was grinded with 300 μL of methanol-1% HCl. The extraction was allowed to occur overnight in darkness and subsequently 200 μL of Milli-Q H_2_0 and 500 μL of chloroform were added to each sample. Anthocyanin was extracted from supernatants of centrifuged samples, using 400 μL of methanol:H_2_0:HCl (60:40:1). The relative amount of anthocyanin was determined using the formula: (*A*_530_–*A*_657_).1000.mg FW^-1^.

### Statistical Analysis

Statistically significant differences were determined based on the Student’s *t*-tests.

## Results

### Assessment of Physiological Parameters Indicative of Plant Drought Stress Status

N7001 and TJS2049 soybean cultivars were compared under optimal conditions or in response to dehydration stress by measuring several physiological and biochemical parameters which are indicative of plant drought stress status. Physiological processes were monitored by means of measurements of accumulated DM, stomatal conductance (SC), and determination of water use efficiency (WUE) and Carbon Isotope Ratio [∂^13^C (‰)]. The percentages of reduction in these parameters values were calculated from measurements under drought stress (35% FC) respect to control plants (100% FC).

**Table 1** shows the magnitude of measured parameters after 6 days of exposure to drought stress. The results showed that drought stress resulted in a lower reduction of all the analyzed physiological parameters in N7001 than in TJS2049. These results are consistent with the field phenotype of N7001, which is a high yielding cultivar that exhibits stable productivity under a variety of environmental conditions (Dr. Sergio Ceretta, personal communication).

**Table 1:** Percentage of reduction of different parameters associated with drought tolerance in the contrasting cultivar.

Parameters	N7001	TJS2049
Accumulated dry matter	50.2	72.1
Stomatal conductance	54.3	75.1
Water use efficiency	26.3	46.3
Carbon isotope ratio	10.4	14.4


*Percentage of reduction for each cultivar from values obtained under drought stress (35% of maximum water retention capacity of substrate) respect to control plants (100% of maximum water retention capacity of substrate).*

**FIGURE 1:**
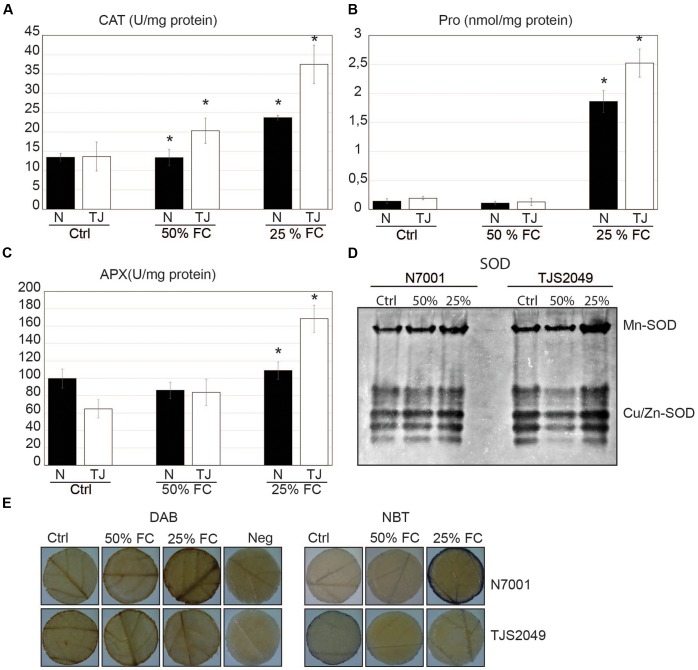
Biochemical responses associated with drought stress status in soybean genotypes. Soybean genotypes: N7001 (N) and TJS2049 (TJ). (Ctrl): Control plants, daily irrigated at maximum field capacity (FC); (50% FC): moderate early dehydration stress; or (25% FC): severe late dehydration stress. **(A)** CAT activity; **(B)** proline levels; **(C)** APX activity in soybean genotypes, grown until V3 and subsequently subjected to moderate stress (3 days without irrigation, 50% FC); or severe dehydration stress (7 days without irrigation: 25% FC). CAT and APX activities are expressed in units per milligram of protein (U/mg). Proline concentration is expressed in nmol per milligram of protein (nmol/mg protein). The values shown are means from one representative technical replicate. Error bars indicate SD (*n* = 10). Three biological replicates were carried out. Significant differences of at least 0.05 confidence level between N7001 and TJS2049 soybean genotypes are marked by asterisks. **(D)** Appearance of SOD activity bands corresponding to different isoforms. Twenty μg of proteins were analyzed in native polyacrylamide gels using in-gel activity assays. **(E)** *In situ* determination of H_2_O_2_ and O_2_ by staining leaf disks with DAB or NBT, respectively. Neg, negative control for DAB staining. Images are the most representative of a pool of five leaf disks per plant/per treatment.

Biochemical parameters, such as proline accumulation and antioxidant enzyme activities, were also addressed to monitor drought stress status. Defense mechanisms against drought-induced oxidative stress were assessed by measuring the activity of enzymes involved in H_2_O_2_ metabolism, like CAT, APX, and SOD, at two different time points after the onset of stress. The first sampling was performed 3 days after the start of the drought stress, where the water retention of the substrate was reduced to 50%. Under these conditions, no visible signs of plant dehydration were observed, and none of the assessed parameters showed changes in N7001, relative to non-stressed control plants. In contrast, TJS2049 responded to these moderate stress conditions by increasing CAT activity (**Figure 1**).

For the second time point, sampling was done 7 days after the onset of plant drought stress. At this time point, water retention of the substrate reached 25% FC, resulting in a more severe stress. All plants exhibited visual signs of stress which were accompanied by an increase in proline content and antioxidant enzyme activity (CAT and APX) (**Figures 1A–C**). TJS2049 also exhibited an increase in SOD activity in contrast to N7001, which displayed activity values similar to the early water stress conditions (**Figure 1D**). Generally, all parameters reached significantly higher values in TJS2049 than in N7001 cultivar.

To investigate whether differences in enzyme activity levels correlated with reduced or increased ROS accumulation in response to stress, leaf disks from control or stressed plants from N7001 and TJ2049 were subjected to DAB staining and NBT staining to detect H_2_O_2_ and superoxide, respectively (**Figure 1E**). Under normal conditions, NBT and DAB staining levels of leaf tissue, tended to be higher in TJS2049 than in N7001, while after 3 days of drought stress, plant leaves from both genotypes did not exhibit significant browning from the different staining procedures, as compared to controls. On the other hand, after 7 days of stress, N7001 displayed enhanced staining levels than TJS2049, suggesting a greater accumulation of ROS in this cultivar under severe stress.

### Characterization of a Subtracted cDNA Library Enriched in Drought-Induced Genes From Soybean cv. N7001

In order to identify genes that were regulated by water deficit in N7001, we constructed a normalized subtracted cDNA library using SSH. Soybean plants were analyzed at an early stress time point (50% FC), and a late stress time point (25% FC).

N7001 plants were grown to V5 stage and RNA samples were extracted from leaves at the indicated time points. For the SSH library construction, samples corresponding to stressed plants at the two different time points were pooled together to define the tester, while samples from well irrigated control plants, taken at the same time points, were pooled to form the driver. PCR products from the forward subtraction process were cloned and 800 randomly chosen clones, with insertions ranging from 250 to 800 bp, were sequenced. The average size of the insert clones was 400 bp. The gene ID of each clone was identified by BLASTn comparisons vs. the genome of *G. max*, cv. William 82 (Schmutz et al., 2010).

Sequence analysis reduced the original set of clones to 740 distinct cDNA sequences, all together representing 390 different genes, most of them (94.4%) showing similarities with genes with known functions.

**FIGURE 2:**
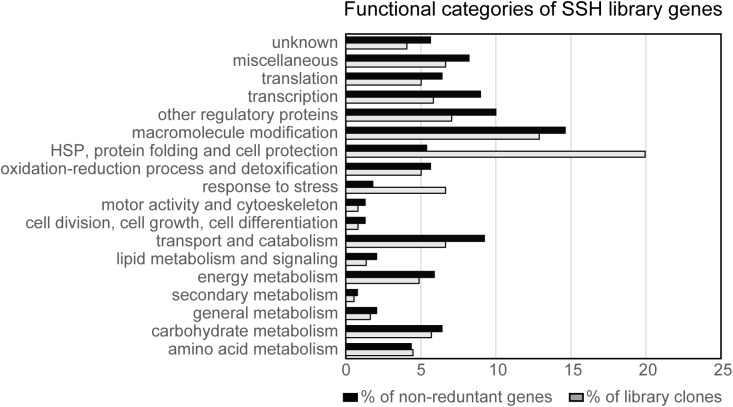
Distribution of library clones and their respective genes into functional categories. The proteins encoded by the N7001-SSH library genes were classified into different categories according to their biological function. In black, percentage of library clones encoding genes from each functional category. In gray, percentage of different genes (non-redundant), belonging to each functional category.

The 390 non-redundant genes were analyzed using Gene Ontology (GO) analysis tool on SoyBase database (Morales et al., 2013). Additional criteria for classification were employed according to protein functional domain annotation or functional experimental evidence existing in the literature. The proteins encoded by the library genes were classified into different categories according to their biological function and involvement in biological process (**Figure 2** and Supplementary Table S1).

When all the library clones were considered in the analysis, genes encoding proteins from the category HSP, protein folding and cell protection, such as LEA proteins, dehydrins and chaperones, had the highest representation, accounting for 20% of the clones. An additional class of genes that was overrepresented in the library was the category of “macromolecule modification,” which represented 13% of the clones and 14.6% of the non-redundant genes. Among the genes included in this category, more than 28% encoded kinases and phosphatases, which together with “other regulatory proteins” were overrepresented in the library.

When the analysis was performed based on the non-redundant genes from the library, the categories involving (i) transcription factors, (ii) macromolecule modification, (iii) other regulatory proteins, and (iv) transport and catabolism, were over represented, accounting for 9, 14, 10, and 9% of the genes, respectively (**Figure 2** and Supplementary Table S1). These analysis also revealed that the categories of “HSP, protein folding and cell protection,” followed by “response to stress,” were approximately four times more represented than when the non-redundant genes were taken into consideration for the analysis, indirectly indicating the high expression levels of these genes.

Genes encoding proteins with putative regulatory or signaling roles belonged to the categories of “transcription,” “translation,” “macromolecule modification,” and “other signaling proteins.” Together, genes falling into these categories represented 40% of the non-redundant genes from the library and 31% of the clones, indicating that enrichment in low abundant transcripts was successful, as these types of genes are usually of low expression.

On the other hand, it was strikingly high the number of clones corresponding to genes that, themselves or their orthologs have been shown to be directly associated with stress responses. These genes accounted for 37% of the library clones and 19% of the genes, indicating that this category had more redundancy in the library than the regulatory/signaling class. The genes belonging to this group encode proteins with a putative direct role in stress responses and included the categories of “HSP, protein folding and cell protection,” “response to stress,” and genes involved in oxidation/reduction processes. Together, these categories comprise protection/repair proteins, and enzymes and structural proteins with a putative role in detoxification or stress adaptation.

### Differential Screening of the cDNA Library Shows Differences Between N7001 and TJS2049 Genotypes in Their Transcriptional Response to Water Deficit

Comparative analysis of the temporal dehydration-induced gene expression was performed by differential screening of the N7001 SSH cDNA library, using forward or reverse subtracted probes derived from N7001 or TJS2049 contrasting cultivars, subjected to 3 or 7 days of water stress. A total of 268 DNA inserts from different clones were separated into three different groups according to their predicted biological function, and arrayed in groups of 96 into cDNA dot blots. Two different clones were placed in more than one location into all the different arrays in order to be able to compare the signal intensity between blots. The first group of clones was composed of genes encoding proteins involved in stress perception, signaling and regulation. This group included genes encoding transcription factors, receptors, kinases, phosphatases and other regulatory genes, among others. A second group consisted of clones representing response genes, such as dehydrins and other LEA proteins, chaperones and genes involved in protection and repair of damaged proteins and membranes. Finally, a third group of clones was composed of genes involved in the production or detoxification of reactive oxygen species, as well as genes encoding proteins of unknown function (Supplementary Table S2). Each 96-cDNA group of clone inserts was blotted eight times onto nylon membranes for hybridization with α ^32^P-labeled forward or reverse subtracted probes from N7001 or TJS2049 genotypes. The probes consisted of drought-induced (forward subtracted) or drought repressed (reverse-subtracted) sequences, at 3 day or 7 day-period after water withdrawal, for monitoring the early or late response, respectively (Supplementary Figure S1).

In order to identify genes that could be associated with genotype specific responses to water deficit, we compared the set of genes that were up-regulated upon stress in N7001 and in TJS2049 cv. We also analyzed each gene set for possible enrichment in specific functional categories. Differences in signal intensities for each clone, between the hybridization with the forward or the subtracted probes, determined whether the gene is likely to be up-regulated (the signal was higher when hybridized to the forward subtracted probe), or down-regulated (the signal was higher when hybridized to the reverse subtracted probe). Expression levels were quantified based on hybridization signal intensity with each probe, using an arbitrary scale shown in Supplementary Figure S1. Each sequence was provided by an arbitrary number reflecting a relative expression value (Supplementary Table S2).

**FIGURE 3:**
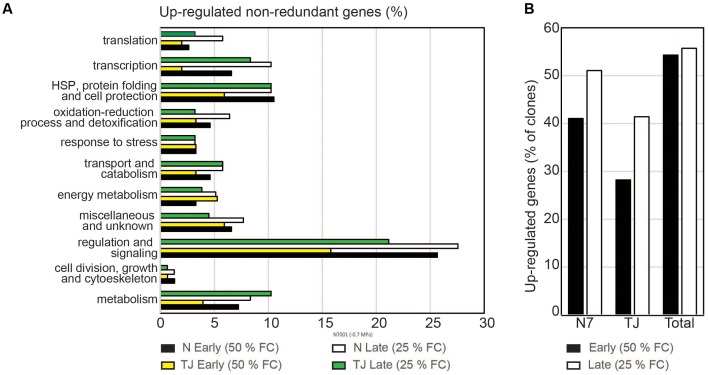
Genotype specific transcriptional responses. **(A)** Percentage of screened genes within each functional category, that were stress-induced in each genotype. Upregulation was determined based on the difference in hybridization signal intensities observed between forward and subtracted probes. Probes derived from N7001 (N) or TJS2049 (TJ) genotypes, at early stress (50% FC) or at late stress (25% FC). Forward subtracted: stress – control. Reverse subtracted: control – stress. **(B)** Percentage of upregulated genes in each soybean genotype, during the early stress response or the late stress response.

Using the criteria of differential hybridization signal intensity, about 54% of the screened genes were found to be up-regulated during the early stress condition in either one or both plant cultivars. A similar proportion (∼56% of the clones) represented genes that were induced during the late stress response (**Figure 3B**).

Screening of cDNA clones with TJS2049 derived probes indicated that the proportion of genes that were up-regulated in response to dehydration in this cultivar was lower than in N7001. This was particularly evident during the early response, at 50% FC, where only 28.2% of the analyzed genes were up-regulated in response to drought in TJS2049, while 40% of the genes were drought induced in N7001. However, the number and type of genes that were up-regulated at an early stage in N7001 was strikingly similar to those that were up-regulated at a late stage in TJS2049, 41% in both cases. On the other hand, certain functional categories of genes were overrepresented in N7001, compared to TJ2049, in particular during the early stress response. The most remarkable differences were found in the number of genes involved in regulation and signaling processes, which were significantly larger in N7001 than in TJS2049 (**Figure 3A** and Supplementary Table S3).

**FIGURE 4:**
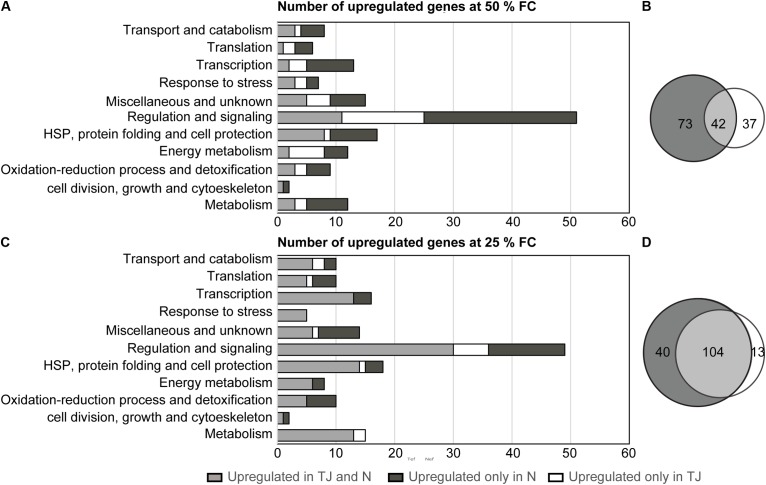
Analysis of overlapping and non-overlapping drought-induced genes in N7001 or TJ2049. Numbers and distribution into functional categories, of clones corresponding to upregulated genes in response to dehydration. **(A)** Moderate early stress (50% FC); **(B)** severe late stress 25% FC). Upregulated genes were selected based on their hybridization signal intensity with specific subtracted cDNA probes. Venn diagram analysis of differentially expressed gene sets indicating the total number of clones corresponding to overlapping and non-overlapping upregulated genes, during early **(C)** or late **(D)** drought stress. N (N7001), TJ (TJS2019).

Dehydration induced gene sets identified in N7001 or TJS2049 were subjected to a Venn diagram analysis to identify overlapping or cultivar-specific drought-induced genes (**Figure 4**). Upon a moderate dehydration stress, out of 152 up-regulated genes, 42 were drought-induced in both cultivars (∼28%), and 37 were specifically up-regulated in TJS2049 (∼24%) (**Figure 4C**). Under this stress condition, a significantly higher number of genes were specifically up-regulated in N7001 cultivar (73 genes, ∼48%). All functional categories, except for genes involved in Energy metabolism, were overrepresented in N7001 when compared to TJS2049 (**Figure 4A** and Supplementary Table S3). In contrast, both cultivars responded to severe dehydration stress by inducing a set of genes of genes in common (104 out of 157), representing ∼67% of all drought induced genes. Under this particular stress condition, only ∼8% of the genes were specifically up-regulated in TJS2049, while ∼25% were specific to the N7001 response (**Figure 4D** and Supplementary Table S4). Similarly, to the early moderate stress response, the N7001-specific up-regulated genes were more represented than TJS2049 specific genes during the late severe stress response (**Figure 4B**). However, no functional category of genes could be associated with a specific cultivar response, supporting that when analyzing this specific set of genes, the two cultivars differ mainly in the time and the intensity level of the transcriptional response.

### Validation of the Differential Gene Expression Profiles

**FIGURE 5:**
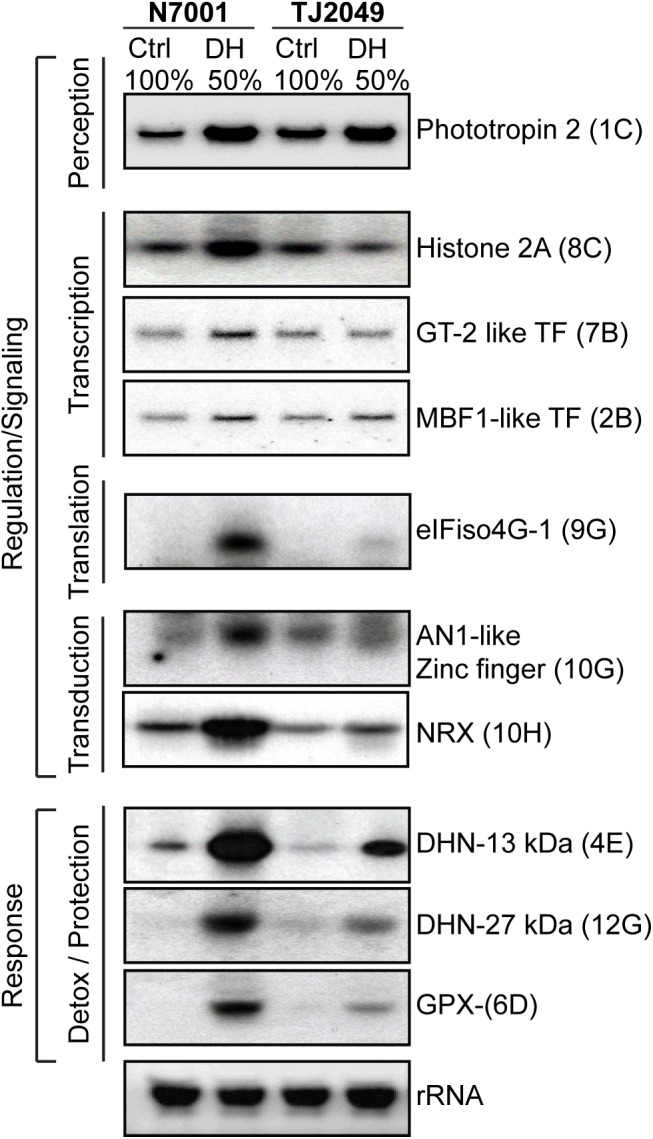
Differential expression of selected genes in response to moderate drought stress in N7001 and TJS2049. Ten μg of total RNA from N7001 or TJS2049 genotypes were analyzed by Northern blot. Controls (Ctrl) correspond to samples from well-irrigated plants, and dehydration (DH) correspond to samples from drought stressed plants, taken when substrate reached a 50% of its water retention capacity (50% FC), usually 3 days after water withdrawal. Clone inserts corresponding to the selected genes were labeled with α ^32^P-dCTP and used as hybridization probes. Ethidium bromide staining of rRNA was used to ensure equal loading of RNA samples. The genes and their accession number used as probes are the following: Phototropin 2 (Glyma.08G264900); Histone 2A (Glyma.15G040400); eIFiso4G (Glyma.17G072500); MBF1-like transcription factor (Glyma.06G276200); GT-2 transcription factor (Glyma.10G225200); AN1-like Zinc finger (Glyma.13G341000); NRX (Glyma.06G021200); Dehydrin: DHN- 13 kDa (Glyma.19G114700); Dehydrin: DHN- 27 kDa (Glyma.09G185500); and Glutathione peroxidase: GPX (Glyma.01G219400). The coordinates showing the position of each clone in the dot blot array (Supplementary Figure S1) are marked in parenthesis.

The expression profile of a group of selected drought-induced genes was confirmed by Northern blot. Since most of the transcriptional differences between the two genotypes were found to occur during the early response to stress, expression analysis of the selected genes was performed 3 days after water withdrawal, at 50% FC (**Figure 5**).

The genes selected for monitoring the early response to drought are associated with transcription or translation regulation, signal transduction and tolerance responses. Seven of them encoded proteins with putative functions in signal transduction or regulation of stress responses. These included a gene encoding phototropin 2, a serine/threonine kinase that functions as blue light photoreceptor; a gene for histone 2A and two genes for transcription factors (MBF1-like and GT-2), all of them regulating transcriptional responses. Other selected genes belonging to the category of signaling and regulation, were eIFiso4G, encoding a plant specific small isoform of eIF4G eukaryotic translation initiation factor; a gene encoding A20/AN1 zinc-finger protein, with a possible regulatory role in stress responses; and a type II nucleoredoxin (NRX2), which is a potential nuclear thioredoxin that may regulate the activity of target proteins in different biological processes.

A glutathione peroxidase (GPX) and two dehydrin encoding genes (DHN-13 kDa and DHN-27 kDa) were selected due to their direct association with stress tolerance processes. GPX may be involved in the regulation of the cellular redox homeostasis, while the DHNs are ubiquitous plant proteins belonging to the group II LEA proteins that accumulate in response to drought and other environmental conditions. (Close, 1997).

All the analyzed genes were strongly upregulated in N7001 in response to drought, and most of them were also drought-induced in TJS2049. Nevertheless, almost all the genes showed higher induction levels in N7001 than in TJS2049 in response to drought (**Figure 5**). This indicates that, although no stress symptoms are observed in plants at 50% FC, these conditions are enough to induce important transcriptional changes in genes involved in signaling and regulation processes, as well as effector genes. The results also show that these changes occur earlier or to a higher extent, in the resistant genotype, N7001, than in TJS2049. The results showed that the expression analysis of the selected genes correlated well with the differential screening data, indicating the overall reliability of the SSH technique. Moreover, the results suggest that the method was successful in selecting over-represented transcripts, including the rare, low abundant, mRNAs, since differences in the expression profiles of several low expression genes, analyzed by Northern blot, could be identified using SSH.

### Genotype-Specific Stress-Dependent Activation of a Gene Controlling Translation Initiation

Given that most significant differences in gene expression between N7001 and TJS2049 plant genotypes occurred during the early response to water deficit, we looked for genes that could have a significant impact on stress tolerance and that were specifically up-regulated in N7001 at an early stage of the response.

A number of genes associated with regulation of protein synthesis were up-regulated upon water deficit. One of these genes that showed a marked difference in expression levels between the two genotypes corresponded to a eukaryotic translation initiation factor eIFiso4G (Glyma.17g072500), designated here as *GmeIFiso4G-1a* for *G. max* eukaryotic initiation factor iso4G-1a. The sequence of this gene was represented in a 516 bp insert clone which contained mostly the 3′UTR of *GmeIFiso4G-1a* (Supplementary Figure S3A).

GmeIFiso4G-1a belongs to a plant specific type of translation initiation factors that exist in addition to the canonical eIF4G factors. Proteins of this type have been shown to interact with eIFiso4E isoforms of the eIF4E translation initiation factors and mediate translation initiation of specific mRNA populations as part of the eIFiso4F complexes (Gallie and Browning, 2001; Mayberry et al., 2009, 2011; Chen et al., 2014).

Cloning of the full length cDNA sequence of *GmeIFiso4G-1a* revealed that this gene encodes an 86.8 kDa protein that, like all members of this protein family, lacks a significant portion of the N-terminal sequence present in canonical eIF4G proteins. GmeIFiso4G-1a has two highly conserved domains, HEAT/MIF4G (from 211 to 457aa) and HEAT/MA3 (from 624 to 746aa) (Supplementary Figure S3B). These domains are present in both eIFiso4G and eIF4G factors and have been shown to be responsible for the interaction with other eIFs during translation initiation (Aravind and Koonin, 2000; Lellis et al., 2010; Gallie, 2016).

A search in the *G. max* genome database Phytozome v12.1, revealed that soybean genome codes for four predicted eIFiso4G proteins and four eIF4Gs. Besides GmeIFiso4G-1a, the other factors of this type were named GmeIFiso4G-1b (Glyma.02G205500), GmeIFiso4G-1c (Glyma.06g225700), and GmeIFiso4G-1d (Glyma.04G154100). The degree of amino acid similarity between GmeIFiso4G-1a and GmeIFiso4G-1b, GmeIFiso4G-1cand GmeIFiso4G-1d deduced proteins was 96, 83, and 75%, respectively.

**FIGURE 6:**
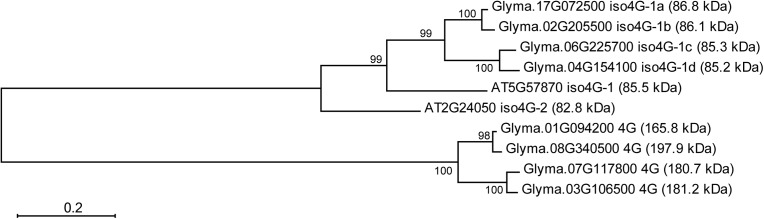
Phylogenetic relationships between eIFiso4G deduced polypeptides of soybean and *Arabidopsis*. Full-length amino acid sequences were aligned by CLUSTAL W and the phylogenetic tree was constructed by the Maximum likelihood method using MEGA6 software. Numbers at branch nodes represent the confidence level of 500 bootstrap replications. The abbreviations of species are as follows: AT, *Arabidopsis thaliana*; Glyma, *Glycine max*. Gene ID, protein name and molecular weight (in kDa) of each sequence are shown to the right of the tree branches. Scale bar represents a distance of 0.2 substitutions per sequence position that occur along the length of the horizontal branches in the tree.

*Arabidopsis* encodes one eIF4G (At3g60240) and two smaller isoforms of eIFiso4G factor, eIFiso4G1 (At5g57870) and eIFiso4G2 (At2g24050) (Lellis et al., 2010). All GmeIFiso4G proteins are more closely related to eIFiso4G1 than to eIFiso4G2 (**Figure 6**).

Cloning and sequencing of the genomic sequence of GmeIso4G-1a, including a 2.4 kb promoter region of from N7001 and from TJ2049 cultivars, showed that no significant differences were found within this gene between the two soybean genotypes or Williams 82 (Supplementary Figure S3A). This indicates that that genotype specific expression of *GmeIFiso4G-1a* observed by Northern analysis, is not dependent on promoter variants.

However, regardless of the genotype, some interesting differences can be appreciated at the promoter regions of Gme*IFiso4G-1a* and its closest homolog, *Gm*e*IFiso4G-1b.* These genes share 81% sequence identity in a 600 bp region upstream from the ATG start codon. Within this region, *GmeIFiso4G-1a* displays differences respect to *GmeIFiso4G-1b* at five blocks located at positions -559 (block 1), -521 (block 2), -478 (block 3), -417 (block 4) and -55 (block 5) in the alignment (Supplementary Figure S3C).

These blocks contain some relevant variants related to stress responsiveness. For instance, block 1 holds a Dof transcription factor binding element and an AAR1 binding element. Dof proteins are versatile transcription factors distinctive of plant lineage. They represent a unique class of transcription factor having bifunctional binding activities, with both DNA and proteins and are expected to participate in the regulation of several process including responses to biotic and abiotic stress (Cai et al., 2013). Block 2 includes another AAR1 binding element and a Heat shock element box, which usually acts cooperatively to increase HS promoter activity (Rieping and Schöffl, 1992). Block 3 contains a SORLIP5AT element, a DRE2 core, a CBF HV site and a LTRE core. SORPLIP element is one of the elements over-represented in light induced promoters and so it is LTRE core (Baker et al., 1994). This last element has been associated with cold and drought-related light-mediated phytochrome signaling (Dubouzet et al., 2003; Jiao et al., 2005). Block 4 contains a GT-1 and an Ibox, both binding sites present in many light regulated gene promoters, and a GATA box, required for high level light regulated and tissue specific expression (Villain et al., 1996; Teakle et al., 2002). Finally, block 5 has a MYC recognition site, related to ABA and cold response (Abe et al., 2003; Agarwal et al., 2006), which is present in *GmeIFiso4G-1b* but not in *GmeIFiso4G-1b* promoter region.

In addition to the differences found among *GmeIFiso4G-1a* and *GmeIFiso4G-1b* proximal promoters regions, these genes harbor many other stress related elements at the variable upstream region of the promoters, including MYC, MYB, WRKY, and Dof binding sites (data not shown).

Starting from the premise that regulation of protein synthesis plays an important role in plant adaptation to stress and considering the genotypic-specific expression profile of *GmeIFiso4G-1a*, this gene was considered a good candidate for conducting further analysis concerning its role in stress tolerance.

### Heterologous Expression of GmeIFiso4G-1a Enhances Stress Tolerance in *Arabidopsis*

Transcript accumulation of *GmeIFiso4G-1a* was only detected in response to dehydration, specifically in the N7001 slow wilting cultivar, suggesting a role for this gene in stress responses.

To gain more insights into the role of GmeIFiso4G-1a, the effect of overexpression of this gene in *Arabidopsis* was assessed by employing an inducible expression system. External control of transgene expression provides a very useful tool for characterization of gene function, since phenotypic impact of a transgenic event can be easily associated to the expression of the transgene. Furthermore, this system prevents possible deleterious effects resulting from constitutive expression of the transgene.

The cDNA sequence of *GmeIFiso4G-1a* was cloned into *pMDC7* binary vector (Curtis and Grossniklaus, 2003) under the regulation of a chemical inducible system (Zuo et al., 2000), which can support high levels of mRNA accumulation upon β-estradiol treatment. Stable transgenic lines were selected for the presence of the gene construct using PCR amplification of the hygromycin resistance gene.

Transgene expression was analyzed in 12 events by Northern blotting after treatment with β-estradiol (Supplementary Figure S2A). *GmeIFiso4G-1a* transcript levels of the selected homozygous T3 lines were confirmed by semi quantitative (RT)-PCR in the presence or absence of β-estradiol (Supplementary Figure S2B). Two homozygous overexpressing (OE) lines, OE-5 and OE-8, showing significant induction levels of the transgene upon β-estradiol treatment, were used for further phenotypic analysis.

**FIGURE 7:**
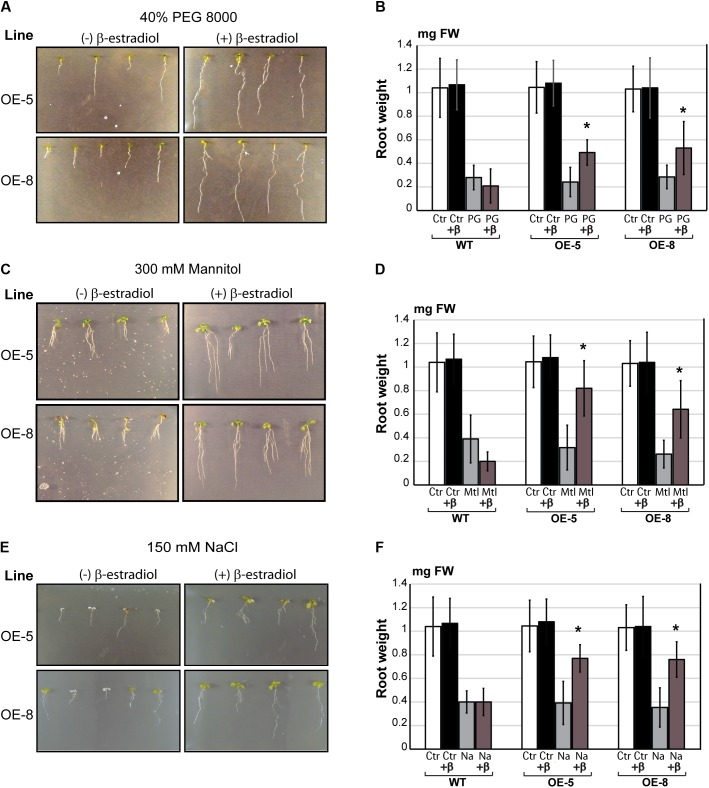
Effect of overexpression of *GmeIFiso4G-1a* on *Arabidopsis* growth under osmotic and salt stress. Five days old seedlings were transferred to high osmotic media: 40% PEG 8000 **(A)** or 300 mM mannitol **(C)**, and to salt stress with NaCl 150 mM **(E)**. Treatments and controls were performed in the presence (+β) or absence of 5 μM β-estradiol, and pictures were taken after 10 days of exposure to stress. Root weight (in mg of fresh weight) of wild-type (WT) and transgenic *Arabidopsis* lines (OE-5 and OE-8), was analyzed in after 10 days of PEG-induced osmotic stress **(B)**; Mannitol-induced osmotic stress **(D)**; or salt stress **(F)**. Controls (Ctrl), 40% PEG 8000 (PG); 300 mM Mannitol (Mtl); 150 mM NaCl (Na). Plants were treated with 5 μM with β-estradiol (+β), or untreated. Asterisk (^∗^) indicates significant differences between the transgenic lines and the wild type at *p* < 0.05 confidence level.

Since the expression of *GmeIFiso4G-1a* in soybean was induced upon drought stress, the phenotype of wild type and transgenic *Arabidopsis* was analyzed in response to drought and various other abiotic stress conditions. To assess whether ectopic overexpression of *GmeIFiso4G-1* can improve plant performance under osmotic stress, *in vitro* grown *Arabidopsis* seedlings were transferred to PEG infused plates or plates supplemented with 400 mM mannitol, and allowed them to grow vertically for up to 9 days. Under these conditions, plants were exposed to final water potential values of ψ ∼ 0.7 MPa, in which plants exhibited a significant decrease of root growth, measured by root weight. Although both wild type and transgenic plants were affected under these conditions, root growth of the wild type was significantly repressed compared to that of the two independent transgenic lines, examined in the presence of β-estradiol (**Figures 7A–D**).

Likewise, improved salt stress tolerance was also observed in transgenic *Arabidopsis* lines overexpressing *GmeIFiso4G-1a*. Root weight was measured in plants growing on vertical plates supplemented with 150 mM NaCl, in the presence or absence of β-estradiol (**Figures 7E,F**). *Arabidopsis* seedlings overexpressing *GmeIFiso4G-1a* displayed longer primary roots than wild type under salt stress in the presence of β-estradiol, suggesting that this gene confers transgenic plants the enhanced tolerance to both osmotic and salt stress.

**FIGURE 8:**
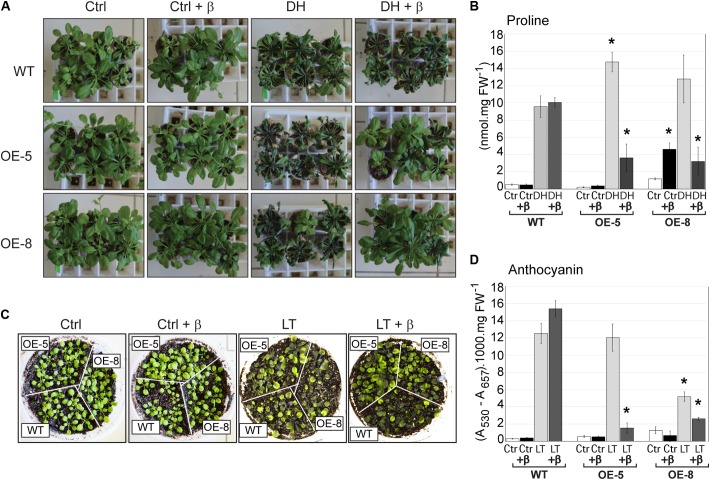
Effect of overexpression of *GmeIFiso4G-1a* on *Arabidopsis* plants exposed to dehydration or low temperature stress. *Arabidopsis* WT or transgenic *GmeIFiso4G-1a* overexpressing lines (OE-5 and OE-8), grown in non-sterile conditions, were analyzed in the presence (+β), or absence of β-estradiol treatment after dehydration or cold stress. **(A)** *Arabidopsis* seedlings were irrigated during 10 days with half strength MS medium. For dehydration stressed samples (DH), irrigation was interrupted almost completely, with the exception of the everyday addition of 200 μL of 5 μM β-estradiol or the same volume of water for non-treated samples. Control (well-watered) plants were treated (+β), or untreated with β-estradiol. Pictures were taken 9 days after the onset of stress. **(B)** Free proline content in well-watered controls (Ctrl) or 9 days dehydration stressed (DH) in the presence or absence of β-estradiol treatment. **(C)** Ten days old *Arabidopsis* plants were exposed to low temperature (4°C) for 2 weeks and photographed. Controls (plants grown at 22°C) and cold stressed plants (LT), treated with of 5 μM β-estradiol (+β), or untreated, were photographed 2 weeks after the onset of stress. **(D)** Anthocyanin content was determined in plants growing at 22°C (Ctrl) or exposed for 2 weeks to 4°C (LT), in the presence (+β) or absence of β-estradiol. Asterisk (^∗^) indicates significant differences between the wild type and the transgenic lines at *p* < 0.05 confidence level.

The performance of wild-type and transgenic plants under drought stress was analyzed in plants growing under non-sterile conditions. Ten days after water withholding, in the absence of β-estradiol treatment, all plants exhibited strong symptoms of wilting. However, treatment of plants with β-estradiol significantly reduced stress symptoms in transgenic plants, but not in the wild type (**Figure 8A**). In line with these findings, proline content of plants exposed to dehydration followed a similar trend in that there was an increase of this amino acid in all plants exhibiting water stress symptoms. Induction of *GmeIFiso4G* by β-estradiol resulted in a significantly lower accumulation of proline in transgenic plants when compared to the wild type (**Figure 8B**).

Similarly, prolonged exposure of plants to low temperature (4°C) resulted in reduced plant growth and high accumulation of anthocyanin in all plant genotypes. However, β-estradiol treatment reduced stress symptoms and anthocyanin accumulation in transgenic lines but not in the wild type (**Figures 8C,D**). Taking together, the results indicate that ectopic overexpression of *GmeIFiso4G-1a* increases plant tolerance to various abiotic stresses.

## Discussion

Drought represents a major constrain in soybean growth and productivity worldwide. Several studies have contributed to the understanding of different aspects of drought stress responses in soybean (Ries et al., 2012; Sadok et al., 2012; Prince et al., 2015; Shin et al., 2015; Tripathi et al., 2016). The introduction of genotypes expressing slow-wilting phenotype into breeding programs has become a promising strategy for the development of drought resistant soybeans (Sloane et al., 1990; Hufstetler et al., 2007; King et al., 2009). However, slow wilting in soybean is a quantitative and multigenic complex trait that is controlled by several quantitative trait loci (QTL) (Abdel-Haleem et al., 2011) and most studies have failed to identify a specific physiological mechanism that explains this phenotype (Ries et al., 2012; Sadok et al., 2012). Therefore, the identification of specific gene targets for improvement of drought resistance is still a valuable current goal for soybean research.

We have analyzed the physiological and molecular responses of two soybean genotypes exhibiting contrasting phenotypes in response to drought stress. In this study, we compared the cultivar N7001, an offspring of the slow wilting PI 416937 (Carter et al., 2003), to the highly drought sensitive TJS2049 cultivar (Pardo et al., 2014). The differences in WUE, SC, carbon isotope ratio and accumulated DM, were used as physiological parameters for assessing drought resistance/tolerance responses. Both in N7001 and TJS2049, dehydration stress resulted in a significant reduction of all the values obtained from the measurements of these parameters. However, compared to TJS2049, N7001 exhibited significantly lower reduction levels of all parameters (**Table 1**), supporting that this cultivar is more resistant to dehydration.

One of the most important quick responses of plants to drought stress is stomatal closure. By closing the stomatal pore the WUE is increased under drought stress conditions (Farooq et al., 2009), thereby reducing the amount of water lost per CO_2_ molecule assimilated. Stomatal conductance is a parameter usually used to determine the rate of diffusion of CO_2_ entering or of water vapor exiting through the stomata. SC decreases during drought stress and the magnitude of decrease is indicative of the extent of plant water stress (Wang, 2012).

Despite SC is a good indicator of plant perception of water deprivation, measurements of this parameter represent a picture of a specific time point and provide no valuable information about longer time lapses. On the other hand, carbon isotopic content (^13^C and ^12^C) is indicative of the accumulated photosynthates during the entire growth period of a plant. Measurements of ^13^C/^12^C in soybean N7001 and TJS2049 showed that both genotypes experienced a significant reduction in carbon isotopic content upon drought stress. This indicates a partial or complete stomatal closure, affecting the diffusion of CO_2_ and H_2_O. Nevertheless, N7001 showed a lower reduction of SC and ^13^C/^12^C parameters than TJS2049, suggesting that N7001 has a better control of water loss, which can be attributed to an enhanced effectiveness of water use (Blum, 2009). Based on these results, it is possible to conclude that the two genotypes are contrasting in drought stress responses, representing valuable plant materials to study the molecular mechanisms involved adaptive responses water deprivation.

Biochemical parameters, including proline accumulation and antioxidant enzyme activities, were addressed to further monitor drought stress status in soybean cultivars. Oxidative stress is a secondary stress in almost any kind of stress, and antioxidant responses are important defenses that can contribute to enhanced stress tolerance. Drought stress causes a cellular buildup of ROS that results in induced changes in enzymatic activities and oxidative damage. The ability of plants to control oxidant levels has been proposed to correlate with stress tolerance (Mittler, 2002; Noctor et al., 2002; Xiong et al., 2002; Jubany-Marí et al., 2010). Among the enzymatic protection mechanism to scavenge ROS, SODs, which can dismutate $O_{2}^{•-}$ into H_2_O_2_, constitute the first line of defense against ROS. The resulting H_2_O_2_ is subsequently detoxified by enzymes like CAT, APX, and glutathione peroxidase (Kar, 2011).

Interestingly, TJS2049 exhibited higher levels of antioxidant enzyme activities (CAT, APX, and SOD), than N7001 upon severe dehydration stress, which was consistent with the lower levels of DAB and NBT staining under these conditions (**Figure 1**). In addition to these observations, proline measurements showed that both cultivars induced proline accumulation in response to severe drought conditions, but TJS2049 accumulated higher levels than N7001. Accumulation of proline is a widespread plant response to environmental stresses such as water deficit and salinity (Szabados and Savouré, 2010; Verslues and Sharma, 2010). Proline is involved in sustaining cellular functions under drought stress by acting as compatible osmolyte and possibly also as a free radical scavenger (Yancey et al., 1982; Samaras et al., 1995; Kaul et al., 2008; Verslues and Sharma, 2010; Signorelli et al., 2014). Nevertheless, a direct correlation between proline levels and stress tolerance has not been clearly established. In fact, in our conditions, proline content may be interpreted as a stress symptom rather than an indication of drought stress resistance. This data is consistent with other studies that show no correlation between high proline content and the level of drought resistance (Ibarra-Caballero et al., 1988). For instance, analysis of proline metabolism in a population of *Arabidopsis* accessions with differences in water stress adaptation, showed a strong association between higher proline levels and accessions that were less adapted to dryer environments (Kesari et al., 2012).

Together, the above data indicate that the mechanisms that underlie improved drought resistance in N7001 are not based on a better protection from oxidative damage through the enhanced capability to scavenge ROS, or on the ability of inducing higher levels of proline.

Large scale transcriptional profiling by RNA-seq and microarrays have provided a great deal of transcriptional information that will facilitate the future the discovery of genes associated with drought stress resistance in soybean. To date, most of the studies involving soybean responses to drought stress by RNA sequencing data or microarrays are available for single genotypes (Guimarães-Dias et al., 2012; Le et al., 2012; Fan et al., 2013; Rodrigues et al., 2015). However, a few studies have provided valuable information by analyzing contrasting soybean genotypes and comparing their transcriptional profile in response to different types of drought stress (Prince et al., 2015; Shin et al., 2015).

In order to contribute to the understanding of the molecular responses of soybean to water deficit, transcriptional responses to drought were analyzed in N7001 cultivar using a normalized subtracted library approach. We thereafter analyzed a subset of genes identified in the subtracted library for differential expression upon dehydration, between N7001 and TJS2049. The goal of this work was to identify candidate genes that could contribute to the phenotypic differences observed between the contrasting genotypes.

Despite the recent advances in gene expression platforms, SSH still has some advantages over other techniques based on next generation sequencing. Besides the lower costs, the construction of cloned cDNA libraries offers the possibility of subsequent screening of the individual clones with different cDNA probes, providing means for further comparisons between different biological samples. Moreover, differential screening of the libraries also provide a way for easily identifying false positive differential expressed genes. One of the most important advantages of SSH in comparison to other RNA profiling methods is the fact that SSH combines normalization and subtraction, resulting in that both abundant and rare transcripts can be equally represented in the library (Cao et al., 2004). This facilitates the detection of rare transcripts, which can be particularly useful for the identification of mRNAs corresponding to genes with regulatory functions at the protein level.

In this study we have identified 390 different genes that were differentially expressed in the slow wilting cultivar N7001 in response to drought stress. Genes involved in regulation, signaling and transduction were highly overrepresented, indicating that library enrichment in sequences corresponding to low abundant transcripts was successful. On the other hand, genes exhibiting the highest degree of redundancy in the library, which is indicative of the expression level, were those encoding proteins with a putative direct role in protection or repair from stress damage (**Figure 2**).

Comparative analysis of a subset of 268 selected library genes using N7001 or TJ2049 subtracted probes allowed the identification of specific genes with differential expression profile between these two genotypes. Overall, the data set indicates that N7001 responds to drought earlier than TJ2049, by up-regulating a larger number of genes upon moderate stress. Genes that were over-represented during the early stress in N7001 compared to TJ2049 belonged to almost all functional categories, with the exception of the category “response to stress,” which had a similar representation in the two genotypes. The categories of genes that showed the most significant differences in the early stress responses between genotypes, included regulatory genes, and genes involved in protection or repair mechanisms (**Figures 3**, **4**). The contrasting genotypes shared fewer differentially expressed genes under moderate than under severe stress. Indeed, upon severe stress, most functional categories were almost equally represented between the two genotypes. This suggests that N7001 responds to stress faster than TJS2049, but in the long term, both cultivars induce similar transcriptional responses upon dehydration. Consistent with these observations, recent work of Shin et al. (2015) showed that the early dehydration-induced transcriptional profile in a slow wilting soybean accession, PI 41937, differed from the response of a drought sensitive cultivar mostly in genes encoding transcription factors or proteins having regulatory functions. This work also showed that comparison of transcriptional profiles between two soybean accessions differing in canopy wilting phenotype showed differences in expression levels at particular time points after stress, and that these differences were principally quantitative rather than qualitative. In this respect, early induced genes are likely to respond to the first signs of water deficit and -at least some of them- are expected to be important for the setup of the defense response. On the other hand, many of the late-induced genes may respond to the physiological consequences of drought stress, by regulating their expression level. In agreement with the results of Shin et al. (2015) and with our data, it is possible to speculate that there is a general transcriptional response to drought stress in soybean, but specific phenotypes such as slow wilting, may rely on the regulation of the level and timing of expression of certain genes.

Since TJS2049 exhibited higher antioxidant enzyme activity than N7001 in response to drought stress, special attention was paid to the category of genes that encoded proteins involved in oxidation/reduction processes and detoxification. Despite the fact that, compared to TJS2049, N7001 showed a larger number of up-regulated genes involved in ROS detoxification, most of the genes from this category were induced in both genotypes (**Figure 4** and Supplementary Tables S3, S4). In addition, the majority of the commonly induced genes from this category exhibited higher induction levels in TJS2049 than in N7001. Moreover, some genes showed a TJS2049 specific drought induction, including catalase (Glyma.04G017500), and chloroplastic Fe-SOD (Glyma.20G196900). These results support the hypothesis that the differences in drought resistance levels between N7001 and TJS2049 genotypes cannot be explained by differences in ROS detoxification abilities, but must rely on other physiological responses.

Despite the difficulty of assigning causal associations between specific genes and phenotypic variations between cultivars, this work was able to identify a number of genes that exhibited a genotypic-specific expression profile in response to drought stress (Supplementary Tables S3, S4). One of these genes (*GmeIFiso4G-1a*) was clearly associated with the response of N7001 to drought stress and encoded a eukaryotic translation initiation factor iso4G (**Figure 5**).

Proteins of this family are plant specific isoforms of translation initiation factors that are part of the cap-binding complex eIF4F. This complex participates in the initial steps in protein synthesis by recruitment of the 40S ribosomal subunit, assisting the recognition of the first codon and promoting assembly of the 80S ribosome (Browning and Bailey-Serres, 2015).

In addition to eIF4F cap-binding complex, which is present in all eukaryotes, plants are unique in that they encode a second complex, named eIFiso4F. These complexes are comprised of distinct isoforms of the cap-binding proteins eIF4E (eIFiso4E), and of the scaffolding protein eIF4G (eIFiso4G) (Browning et al., 1992; Browning, 1996). Within this complex, eIF4G or eIFiso4G interact with other factors, such as eIF4A, eIF4B and eIF5 and the poly(A) binding protein (PABP), allowing mRNA recircularization and recruitment of the preinitiation complex 43S (PIC) to the mRNA (Gallie, 2014; Browning and Bailey-Serres, 2015; Hinnebusch et al., 2016; Sesma et al., 2017). Structurally, the eIFiso4G isoform differs from the canonical eIF4G in that the first one is a significantly smaller protein due to the lack of a large portion of the N-terminal sequence that is present in eIF4G (Browning et al., 1992). Nevertheless, eIFiso4G is highly conserved in the MIF4G and MA3 HEAT domains, which are responsible for the binding to eIF4E (or eIFiso4E), eIF4A and eIF3. Although both type of factors are capable of initiating translation, *in vivo* and *in vitro* studies in *Arabidopsis*, have shown that there is a functional specialization among eIF4G and eIFiso4G (Gallie and Browning, 2001; Cheng and Gallie, 2010; Lellis et al., 2010; Chen et al., 2014; Gallie, 2016).

GmeIFiso4G-1a is one of four members of this protein family in soybean. Phylogenetic analysis revealed that GmeIFiso4G-1a and 1b are extremely similar proteins (96% identity) and GmeIFiso4G-1c and 1d are also almost identical to each other, suggesting that in both cases, these are likely to represent gene duplication events. Nevertheless, all four members of this class fall into one group together with the eIFiso4G1 isoform from *Arabidopsis* (**Figure 6**). This is consistent with the results of Gallie (2016), who proposed that eIFiso4G1 isoform is present in all plant species, while eIFiso4G2 is specific for the Brassicaceae. The same work also demonstrated that eIFiso4G1 and eIFiso4G2 are functionally different in supporting translation of certain mRNAs. In fact, eIFiso4G2 appeared to be more similar to eIF4G than to eIFiso4G1 in transcript selectiveness, in particular when mRNAs containing the virus-derived W 5′ leader were taken into account (Gallie, 2016).

The number of genes encoding eIFiso4G1 orthologs varies between plant species and functional specialization among this group has not been assessed. Although it is not clear whether the distinct members of soybean eIFiso4G proteins have functional differences between each other, the fact that only *GmeIFiso4G-1a* was identified in the subtracted library suggests that there might be a differential regulation among the expression of these genes. However, this is just merely speculative, since in this work, *GmeIFiso4G-1a* transcript accumulation in soybean was assessed by Northern blot using a 560 bp probe from an SSH clone insert (Supplementary Figure S3B), which shared high sequence identity at the nucleotide level with other *eIFiso4G* genes: 100% to *GmeIFiso4G-1a*; 96% to *GmeIFiso4G-1b*; 79% *to GmeIFiso4G-1c*; and 80% to *GmeIFiso4G-1d*. Therefore, it is likely that cross-hybridization occurred between the cDNA clone sequence and other *eIFiso4G* genes, at least with *GmeIFiso4G-1b*.

Analysis of promoter sequences of *Gm*e*IFiso4G* genes showed no significant similarities between *GmeIFiso4G-1a* and *GmeIFiso4G-1c* or *GmeIFiso4G-1d*. In contrast, *GmeIFiso4G-1a* and *GmeIFiso4G-1b* share a high sequence identity in the first 600 bp region upstream from the initiation codon (Supplementary Figure S3C), suggesting that expression of these two genes may be equally regulated. Nevertheless, these genes exhibit some differences involving the presence or absence of certain elements, such as Dof, DRE, CBF, and MYC sites, arguing in support of the idea that the members of the *eIFiso4G* gene family are specifically regulated at the transcriptional level.

Interestingly, neither *GmeIFiso4G-1a* nor the other soybean *eIFiso4G* genes, were among the upregulated genes that were identified upon drought stress in the different transcriptome datasets consulted (Le et al., 2012; Rodrigues et al., 2015), supporting the genotypic specificity of the expression pattern of *GmeIFiso4G-1*.

The role of eIFiso4G factors in plant stress responses has been poorly addressed. In this work, we have evaluated the function of *GmeIFiso4G-1a* in abiotic stress tolerance by ectopic expression of the gene in *Arabidopsis*. We used a strategy of conditional overexpression of the transgene to permit temporal control of gene expression. When exposed to osmotic stress (PEG or Mannitol), salinity (NaCl), dehydration or low temperature, transgenic plants overexpressing *GmeIFiso4G-1a* performed significantly better than the same plants in non-inductive conditions or than the wild (**Figures 7**, **8**). Furthermore, drought induced proline accumulation and cold-induced anthocyanin synthesis in transgenic plants were lower under transgene induction conditions than under no induction conditions or than the wild type, suggesting that overexpression of *GmeIFiso4G-1a* reduces stress levels in *Arabidopsis*.

Other evidences that point out the importance of *eIFiso4G* genes on plant stress responses came from the phenotypic analysis of loss of function *eIFiso4g1/2* single and double *Arabidopsis* mutants. This work showed that the loss of both forms results in a severe growth phenotype, changes in chlorophyll levels and impaired responses to heat or salinity (Lellis et al., 2010). Further studies indicated that eIFiso4G1 and eIFiso4G2 are required in *Arabidopsis* to support photosynthetic activity and plant growth, and that this effect, at least partially, is due to the regulation of the expression of a gene involved in the xanthophyll cycle, encoding violaxanthin de-epoxidase, or VDE (Chen et al., 2014). Interestingly, knockout mutants showed a misregulation of VDE expression both at the translational and transcriptional level (Gallie, 2016). We did not detect major differences in the photosynthetic activity or related parameters of the transgenic *GmeIFiso4G-1a* overexpressing lines (data not shown). However, a “gain-of-function” overexpression approach is not necessarily expected to lead to opposite phenotypes than the “loss-of-function” strategy based on the analysis of null mutants. Further analysis has to be carried out for a deeper understanding of *GmeIFiso4G-1* mode of action.

The regulation of translation plays a key role in plant adaptation to environmental stress by globally modulating gene expression, as well as by modulating specific genes. A number of studies demonstrate that different abiotic stresses, such as hypoxia, light, salinity, heat, and dehydration, result in a general inhibition of protein synthesis (Kawaguchi et al., 2004; Branco-Price et al., 2005, 2008; Kawaguchi and Bailey-Serres, 2005; Floris et al., 2009; Mustroph et al., 2009; Matsuura et al., 2010; Juntawong and Bailey-Serres, 2012; Liu et al., 2012; Muñoz and Castellano, 2012; Yángüez et al., 2013). While translation inhibition upon stress reduces energy consumption, certain specific mRNAs are selectively translated to produce relevant proteins involved in the proper establishment of the stress adaptation process. Supporting this statement, a recent work focusing in heat stress, showed that under this condition, the translation of the majority of mRNAs was reduced approximately 50%, while a group of mRNAs detected by polysome profiling, were able to maintain high translation rates (Yángüez et al., 2013). These specific mRNAs coded for regulatory and effector proteins involved in drought, ion, salt or wounding stress, reinforcing the importance of translational regulation in stress adaptation. Even though, the mechanisms by which some mRNAs are sensitive or recalcitrant to the global inhibition of translation upon stress are not fully understood, evidences support the idea that selectiveness is partially defined by specific features within the mRNA sequences (Yángüez et al., 2013; Gallie, 2016). It is expected that the progress in this field of research will have a significant input in improvement of stress tolerance in crops by contributing to the development of new strategies based on the regulation of relevant protein synthesis. Since eIFiso4G type of factors are unique to plant kingdom, it is expected that they participate in the regulation of translation of proteins implicated in plant specific processes, like those related to photosynthesis, flowering or stress responses, which makes them excellent candidates to pursue that goal.

## Author Contributions

JG performed the SSH library, generated and analyzed the *Arabidopsis* transgenic plants. CR contributed to the soybean characterization and *Arabidopsis* phenotyping. EC performed soybean phenotyping. AF contributed to *Arabidopsis* characterization and revising the manuscript. VB contributed to the library analysis and provided suggestions. OB supervised the soybean characterization, provided suggestions, and revised the manuscript. SV conceived and directed this study, analyzed the data, and wrote the manuscript. All authors approved the manuscript and the version to be published, and agreed to be accountable for all aspects of the work in ensuring that questions related to the accuracy or integrity of any part of the work are appropriately investigated.

## Conflict of Interest Statement

The authors declare that the research was conducted in the absence of any commercial or financial relationships that could be construed as a potential conflict of interest.

## Footnotes

1 https://phytozome.jgi.doe.gov
