# Supplementary material for: A Dehydration-Induced Eukaryotic Translation Initiation Factor iso4G Identified in a Slow Wilting Soybean Cultivar Enhances Abiotic Stress Tolerance in Arabidopsis
Source: Front Plant Sci. 2018 Mar 2;9:262. doi: 10.3389/fpls.2018.00262 (PMC5840855; doi:10.3389/fpls.2018.00262)

Supplementary Material

A dehydration-induced eukaryotic translation initiation factor iso4G from a slow wilting soybean cultivar enhances abiotic stress tolerance in *Arabidopsis*

Juan Pablo Gallino, Cecilia Ruibal, Esteban Casaretto, Andrea L. Fleitas, Victoria Bonnecarrère, Omar Borsani, Sabina Vidal*

*** Correspondence:** Sabina Vidal: svidal@fcien.edu.uy

Figure S1. Differential screening of representative clones from SSH by reverse Northern blots. Dot blots of forward subtracted cDNA library clones were hybridized with [α ^{32}P] dCTP labeled forward or reverse subtracted probes synthesized from RNA samples of N7001 or TJS2049, subjected to 3 days without irrigation (50 % field capacity-FC) or 7 days (25 % FC), to monitor the early or late response, respectively. Two hundred and eighty cDNA clones were grouped into arrays of 96 clones according to their putative function in: signaling and regulation; response (protection/repair); and a third group that included detoxification and other proteins. The position of the clones selected for further expression analysis is marked by a dashed circle. *GmeIFiso4G- 1a* coding sequence is indicated with an asterisk.

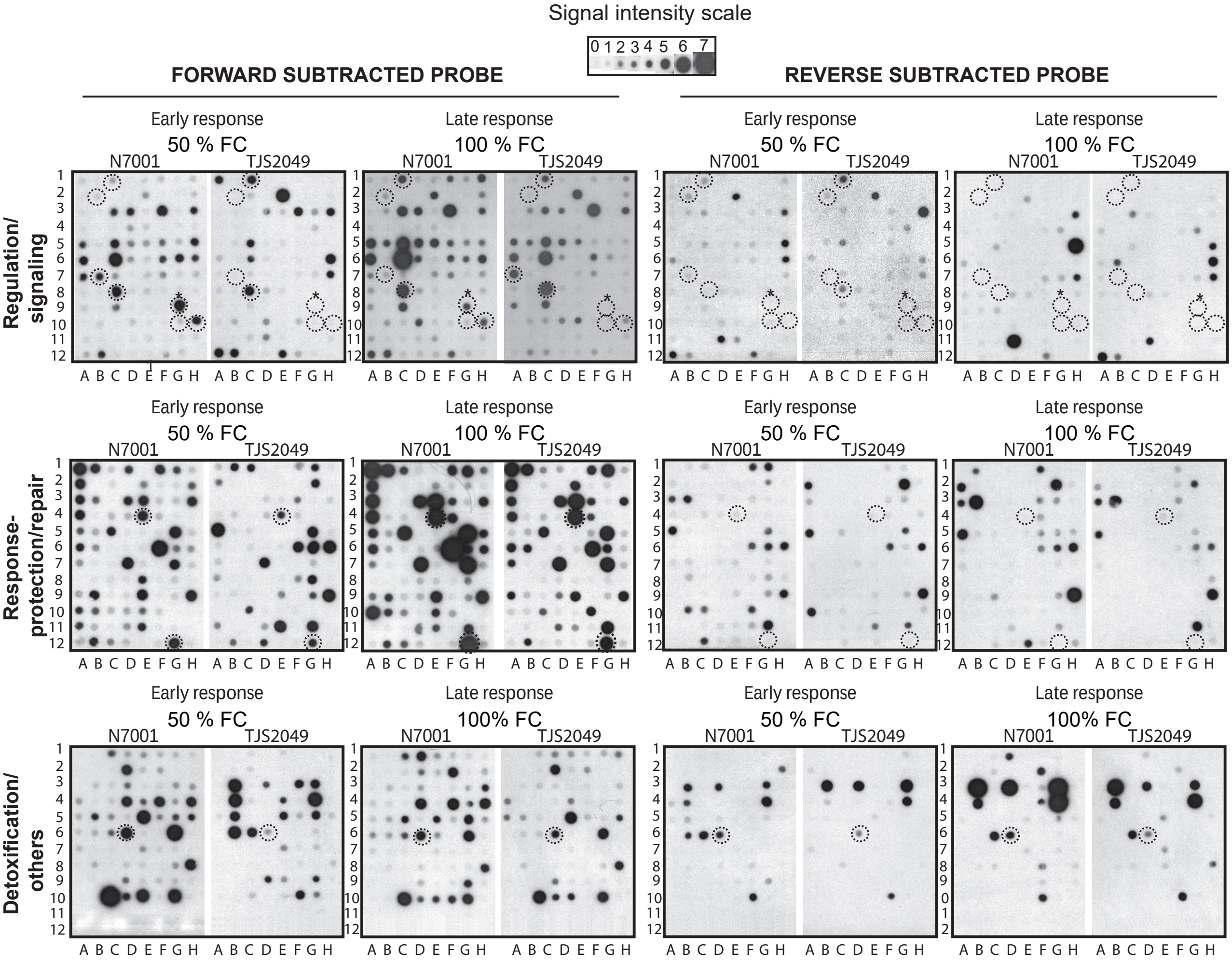

Figure S2. Transgene expression in transgenic *Arabidopsis* lines carrying *GmeIFiso4G-1a* under the control of β-estradiol inducible promoter. **(A)** Northern blot analysis of the expression levels of *GmeIFiso4G-1a* in response to β-estradiol treatment. Two weeks old transgenic T2 *Arabidopsis* lines, grown in the absence of the inducer, were treated with 5 μM β-estradiol and sampled 24 h after treatment for RNA extraction. Eight micrograms of total RNA were blotted onto a nylon membrane and hybridized with [α ^{32}P]- dCTP labeled full-length cDNA of *GmeIFiso4G-1a*. Ethidium bromide staining of rRNA was used to ensure equal loading of RNA samples. Twelve independent events are shown. **(B)** Selected overexpression lines (OE-5 and OE-8), were further analyzed in the presence or absence of 5 μM β-estradiol treatment, by RT-PCR amplification of *GmeIFiso4G-1a. Arabidopsis* Ubiquitin gene (At4G05320) was used as an internal control for cDNA levels. WT: wild-type plants; OE-5 and OE-8: two different transgenic overexpressing lines; + β: 24 h treatment with 5 μM β-estradiol.

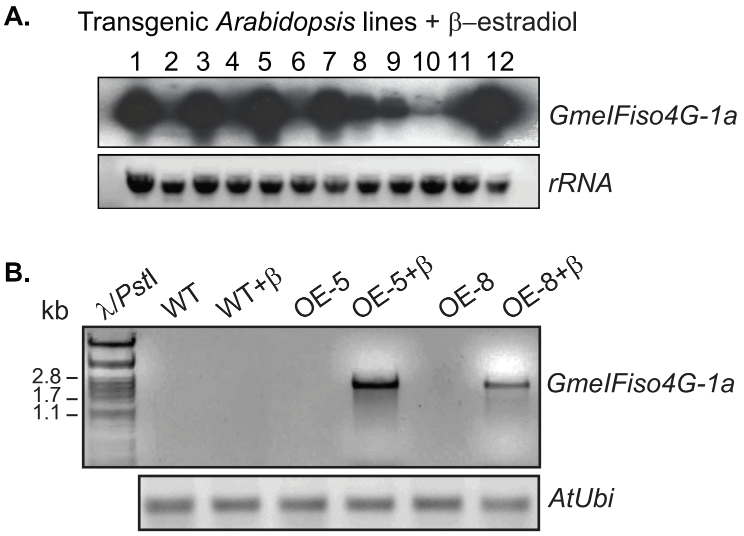

Figure S3. (A) Alignment of nucleotide sequences of SSH clone and *GmeIFiso4G* transcript sequences. *GmeIFiso4G-1a* cDNA sequence from N7001 cv. was compared to the four transcript sequences from soybean *eIFiso4G* of Williams 82 cv. The initiation and termination codons for the ORFs are boxed and marked above the alignment with an asterisk.

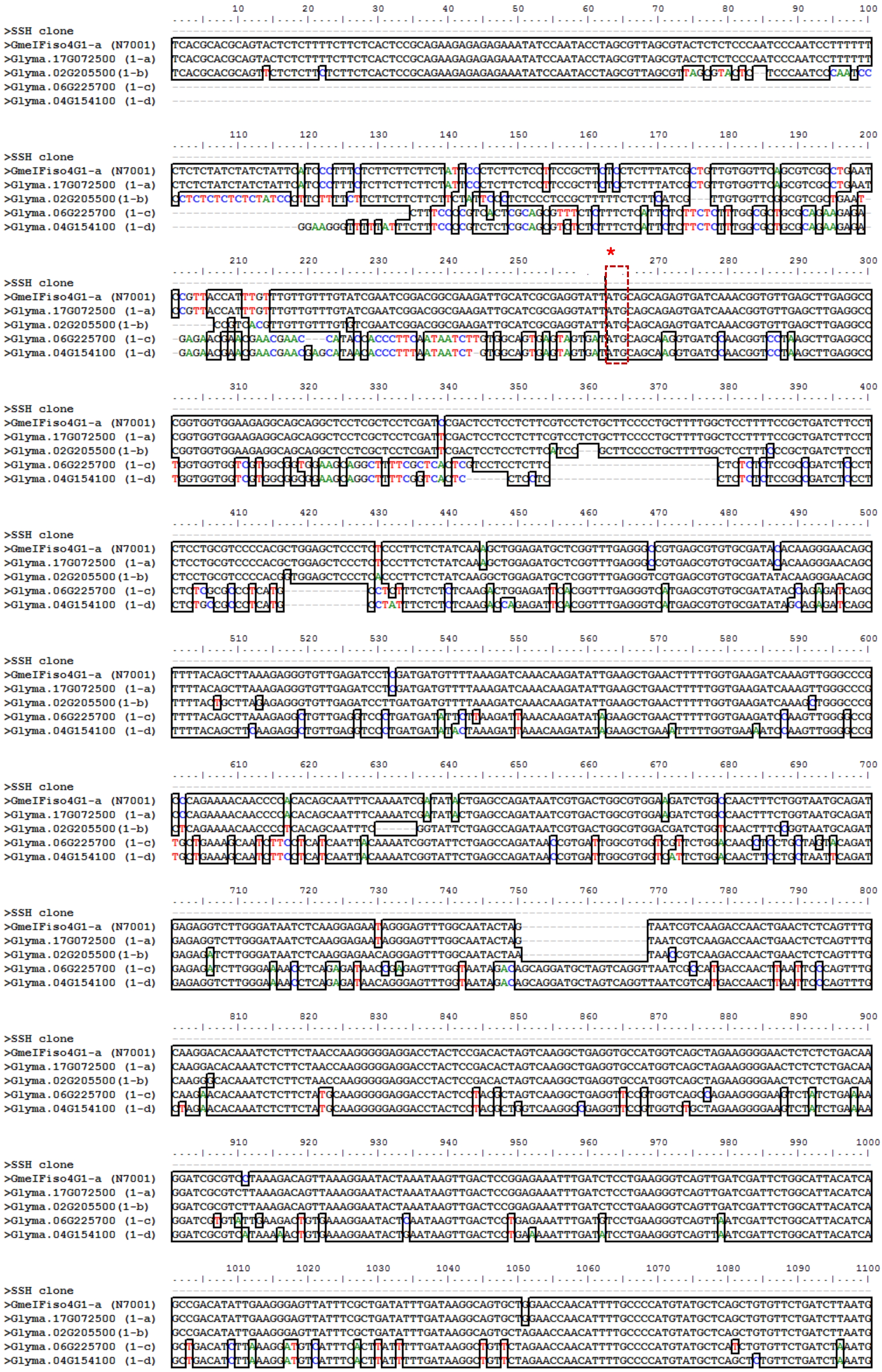

>SSH clone
>GmeIFiso4G1-a (N7001)
>Glyma.17G072500 (1-a)
>Glyma.02G205500(1-b)
>Glyma.06G225700 (1-c)
>Glyma.04G154100 (1-d)

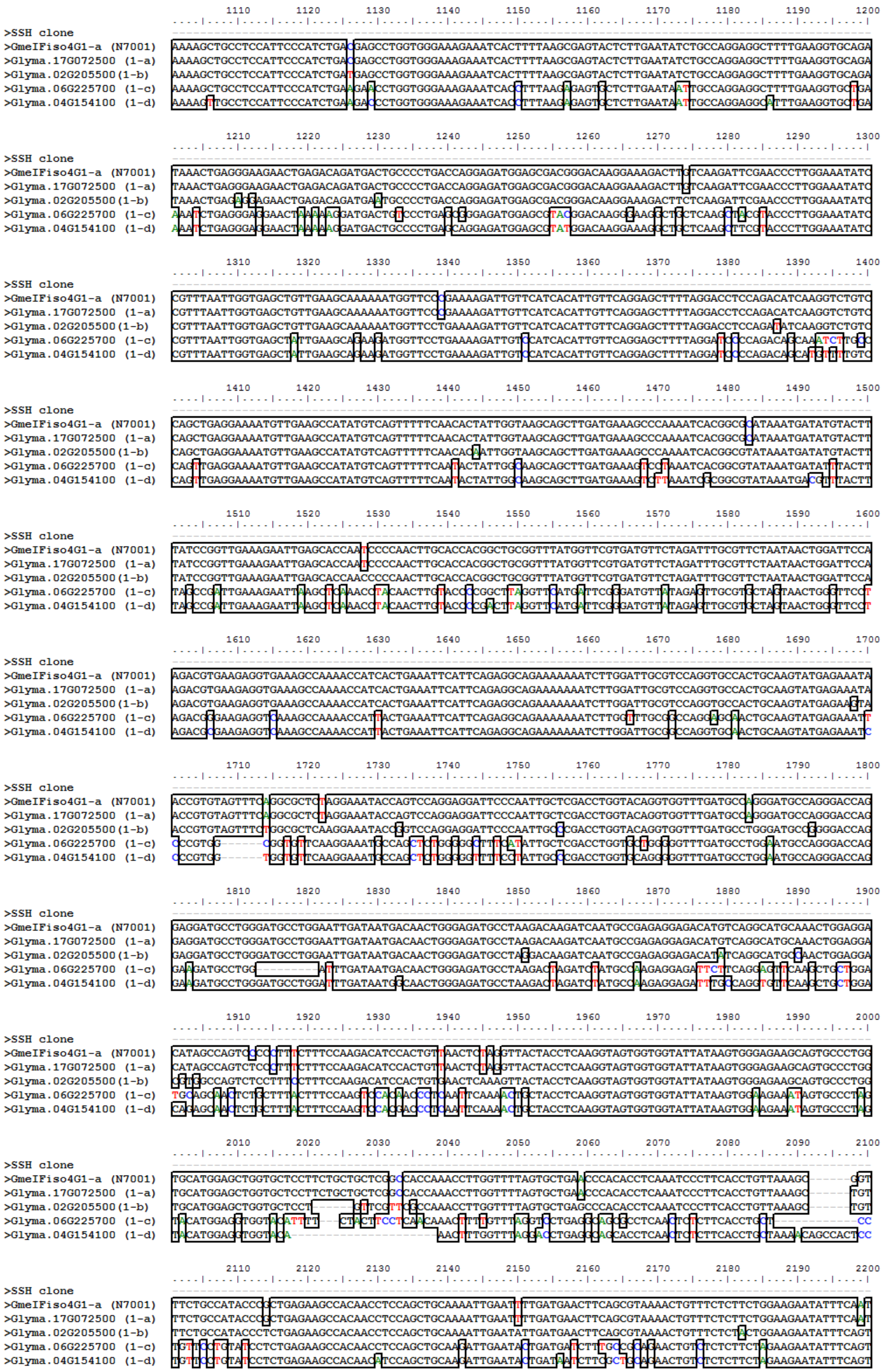
>SSH clone
>GmeIFiso4G1-a (N7001)
>Glyma.17G072500 (1-a)
>Glyma.02G205500 (1-b)
>Glyma.06G225700 (1-c)
>Glyma.04G154100 (1-d)

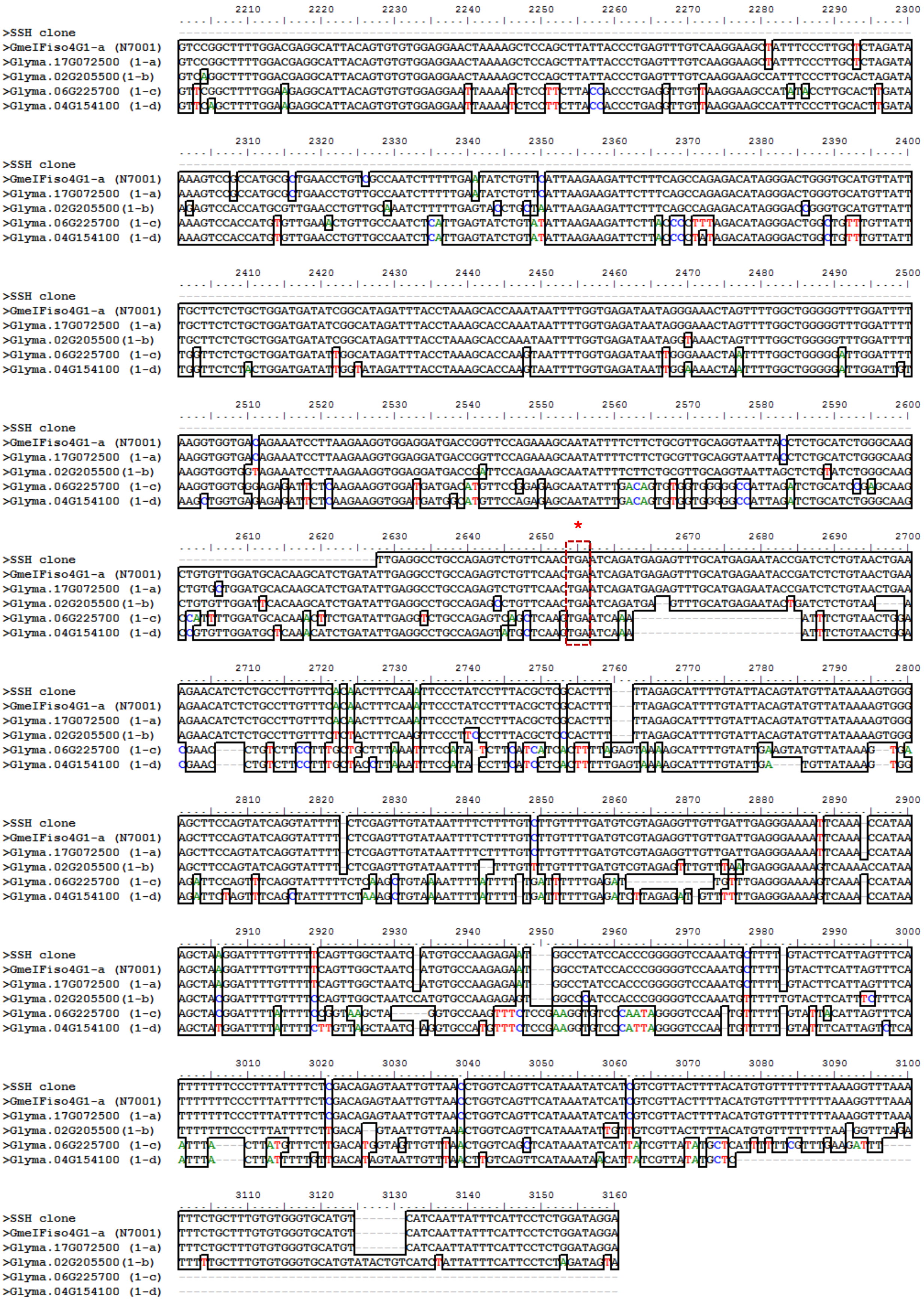
>SSH clone
>GmeIFiso4G1-a (N7001)
>Glyma.17G072500 (1-a)
>Glyma.02G205500 (1-b)
>Glyma.06G225700 (1-c)
>Glyma.04G154100 (1-d)

Figure S3. (B) Schematic representation of GmeIFiso4G-1a protein, coding sequence (CDS) and the insert sequence of the SSH hybridization clone, which was used as hybridization probe for Northern analysis. MIF4G and MA3 domains are indicated in the protein. Nucleotide positions of the start and finalization of the probe sequence are shown in the transcript sequence.

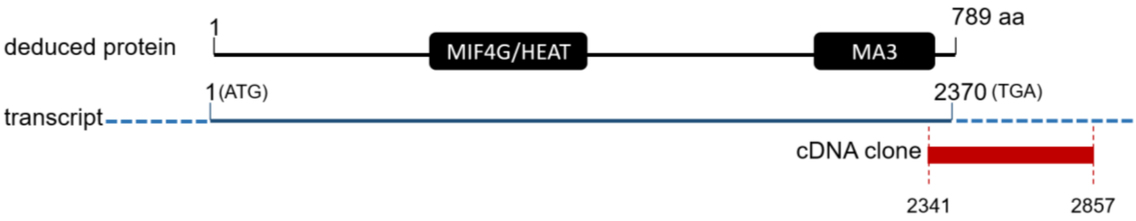

Figure S3. (C) Schematic representation of differences between promoter elements from soybean *GmeIFiso4G-1a* and *GmeIFiso4G-1b*. In silico analysis of the highly homologous 600 bp region upstream of the start codon of the genes (+1) was performed using PLACE online resource (Higo et al., 1999). Block 1: Dof 1 and AAR1 elements; Block 2: AAR1 and heat shock elements; Block 3: SORLIP5AT, DRE2 core, CBF-HV, LTRE core elements; Block 4: GT-1, Ibox, GATA box elements and Block 5: MYC consensus element.

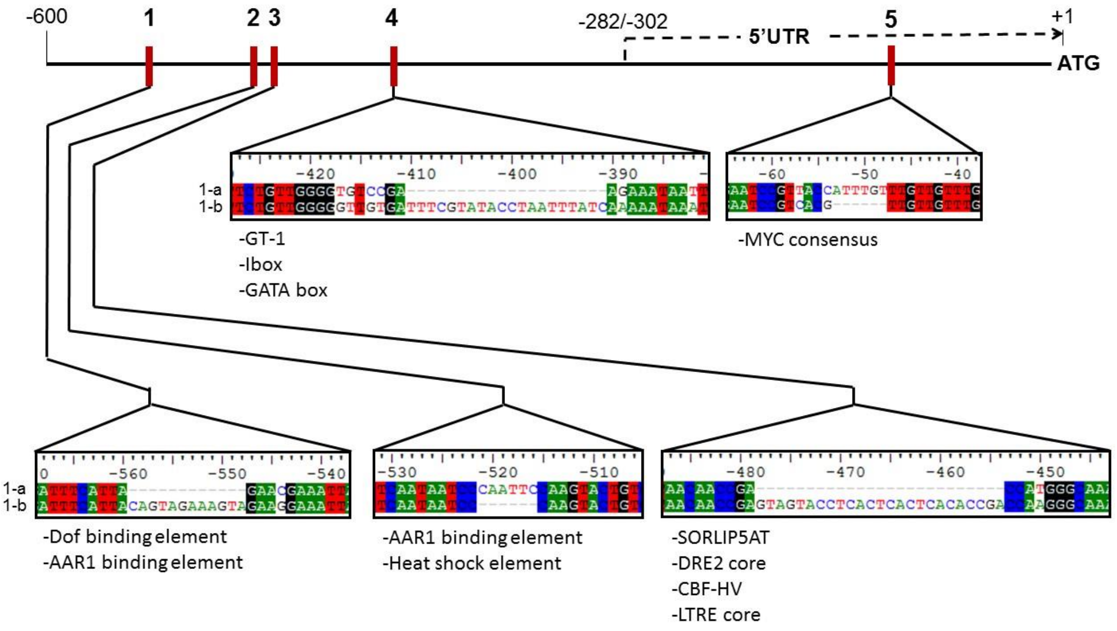

Supplement: Supplementary file 1 [file Presentation_1.pdf]
